# Three-Dimensional CFD Simulations for Characterization of a Rectangular Bubble Column with a Unique Gas Distributor Operating at Extremely Low Superficial Gas Velocities

**DOI:** 10.3390/mi17020191

**Published:** 2026-01-30

**Authors:** Arijit Ganguli, Vishal Rasaniya, Anamika Maurya

**Affiliations:** School of Engineering and Applied Sciences, Ahmedabad University, Ahmedabad 380005, Gujarat, India; vishal.r@ahduni.edu.in (V.R.); anamika.maurya@ahduni.edu.in (A.M.)

**Keywords:** bubble columns, CFD simulation, flow patterns, bubble plume, gas holdup, microbubbles

## Abstract

In the present work, three-dimensional (3D) simulations have been performed for the characterization of a rectangular column for a uniform gas distributor with µm-sized holes at a ratio of 5. The model is first validated with experimental data from the literature. Simulations are then performed for a gas distributor with identical pitch but two different hole sizes, namely 600 µm and 200 µm. Three superficial gas velocities, namely 0.002 m/s, 0.004 m/s, and 0.006 m/s, were used for each distributor type. The gas movement in the fluid is found to be a strong function of hole size. For a 600 µm hole size, the operating condition has minimal impact on gas plume movement and moves centrally in a fully aerated regime. However, for a hole size of 200 µm, for all superficial velocities, the gas plume movement is dynamic and partially aerated. The plume moves along the right wall initially and then follows vertically. These characteristics are different from the meandering plume in centrally located spargers. The liquid mixing in the bulk is a function of time. During the plume development flow, different shapes are observed. Based on the analogy with the shapes found in nature, these shapes have been termed as balloon, cap, jet or candle flame, bull horn, mushroom, tree shape, and disintegrated mushroom shapes. Quantitative insights have been obtained in the form of time-averaged radial profiles of both volume fractions and liquid axial velocities. A symmetric parabolic shape for a hole size of 600 µm and skewed asymmetric shapes for a 200 µm hole size for three different axial positions, namely 0.1, 0.25, and 0.4 m, are observed. Correlations for gas holdup and liquid velocity have been proposed for low superficial velocities, which are in good agreement with the CFD simulation data, with a deviation of 15–20%. The deviations are partly due to the use of the k-ε turbulent model. The correlations perform better than the correlations available in the reported literature for similar superficial gas velocities.

## 1. Introduction

Bubble columns have been studied for equipment that is used in chemical and petrochemical industries. However, this equipment has high potential to serve in biochemical reactions if operated at extremely low velocities (less than 0.01 m/s).

**Figure 1:**
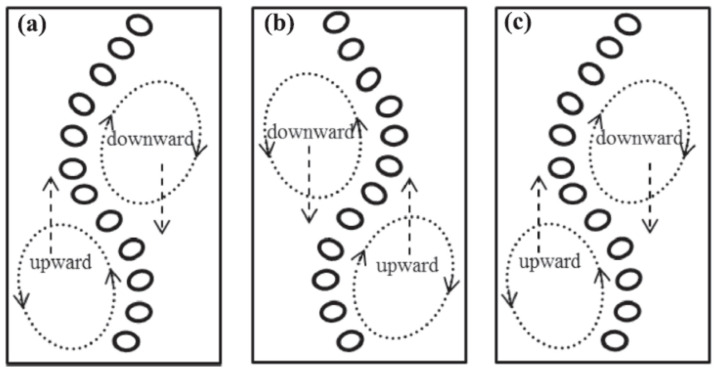
Oscillation of a bubble plume (**a**) Stage 1, (**b**) Stage 2, and (**c**) Stage 3 (Reproduced with permission from Liu, Yan, Ziegenhein, Hessenkemper, Li, and Lucas [5]).

In these columns, the gas disperses into a fluid and rises in the form of a plume, which depends on various factors. Like geometric and operating parameters and the fluid properties. These columns are generally characterized by homogeneous and heterogeneous regimes. Operating parameters include superficial gas velocity, operating temperature, and pressure, while geometric parameters include column diameter, distributor geometry, aspect ratio, and fluid properties include density, viscosity, and surface tension [1,2]. The location of the gas distributor may be centrally located with holes concentrated in only 20% of the area or uniformly distributed in the plate area. The gas dispersed into the liquid may rise uniformly in the case of a uniform distributor or as a meandering plume in the case of a centrally located distributor, as shown in Figure 1.

As can be observed, the plume does not follow a straight path but is characterized by a meandering bubble region surrounded by a staggered arrangement of flow vortices in the liquid phase, causing instability (mostly Rayleigh–Taylor instability) in the column. One cycle in the plume was achieved from stages 1–2–3 (where 1 represents the initial position aligned to the left, 2 represents the plume moved to the right, and 3 represents the plume back to its initial position at the left). The circulation cells move upward and downward shown by the upward and downward arrow respectively in Figure 1. The cyclic process continues with time, and the number of plume oscillating cycles (*n*) and corresponding time period (T) were generally obtained by several researchers [1,2,3,4,5] by visual observation and manual counting. The frequency (calculated as (*n*/T)) depended on the selection of *n* in such a way that the value of T does not change with time for further cycles. Two important dimensionless numbers that determine the dynamic characteristics of the bubbles are the modified Strouhal number ($\frac{fL_{i}}{U_{G}}$) and the Rayleigh number ($\frac{\rho gL_{i}^{2}U_{G}}{\mu U_{B}^{2}}$). The Strouhal number is a dimensionless number describing oscillating flow mechanisms using the frequency (*f*) of bubble plumes, while the Rayleigh number defines the convective flows due to the frequency of small bubbles [5]. *U*_G_ and *U_B_* represent the superficial gas velocity and bubble rise velocity, while *L_i_* represents the initial plume width equal to the distributor area. The knowledge of plume oscillation due to centrally located spargers and meandering plumes, the dimensionless numbers and frequency, is important for characterizing other plume types. The movement of the bubble plume (meandering back and forth) causes movement of vertical structures in the liquid (continuous phase), which determines the amount of liquid mixing. This mixing at the local level is mostly turbulent, giving rise to overall mixing and better reactor performance. The local flow turbulence and gas holdup distribution dictate the performance of the bubble columns due to their complex relationship. Hence, it is important to understand the dynamic characteristics of BCRs by performing numerical simulations and analyzing the transient mixing characteristics. Researchers [6,7] have also shown that vortical structures are not limited to higher superficial velocities but can also be formed at low superficial velocities due to the type of the gas distributor [8,9]. In numerical investigations, 2D BCs were initially studied using Computational Fluid Dynamics (CFD). CFD of BCs have been analyzed computationally using two approaches, namely the Eulerian–Eulerian (EE) or Eulerian–Lagrangian (EL) methods. In EL models, the governing equations for each bubble are coupled with the liquid phase governing equations. In EE models, both gas and liquid phases are treated as interpenetrating continua. The dispersed phase (gas) is considered to be a pseudo-continuum and represents a single bubble size in the closures of various interfacial forces, such as drag, and would represent a single advection phase velocity. The forces apart from the drag force that affect the overall fluid motion are termed the non-drag forces. The interfacial non-drag forces are the lift force, the turbulent dispersion force, the virtual mass force, and the wall lubrication force. Masood and Delgado [10] carried out numerical studies on BCs operated at low gas velocities and found that for low gas velocities of around 0.005 m/s, the drag given by the Schiller and Naumann correlation [11] predicted the gas holdup well. However, the authors found that the RNG k-ε model renormalizes the NS equations to account for the turbulent diffusion for all different scales of motion. Four different drag laws were tested, and all of them were able to predict the axial liquid velocity accurately. The authors also carried out sensitivity studies to understand whether the combination of Drag-Lift (DL) models, and Drag-Lift-Turbulent-Dispersion (D-L-TD) models, Drag-Lift-Turbulent-Dispersion-Wall Lubrication force-Virtual Mass force (D-L-TD-WL-VM) was able to predict the right trends and magnitudes of axial velocity profiles when compared to experimental data. The authors found that in some cases the DL predicted the axial liquid velocity profiles accurately, while in some cases the D-L-TD-WL-VM combination was needed.

Table 1, Table 2 and Table 3 summarize a few relevant experimental and numerical works carried out by different authors. Since centrally located distributors with meandering plumes have been well studied numerically.

Becker, De Bie, and Sweeney [3] performed numerical simulations in BCs and found that the hydrodynamics were characterized by a gross circulation flow with upward flow in the center and downward flow along the walls for the time-averaged liquid phase flow. The instantaneous flow structures were marked by the irregular axial movement of vortical structures through the column [6]. Experimental investigations in the heterogeneous regime have also shown that the time-dependent flow in a periodic bubble column was chaotic and not predictable due to the radial movement of the bubbles in the distributor zone [7]. Further, the low-frequency oscillations of the axial and tangential velocities have significant amplitudes in any position of the column. Pfleger et al. [12] carried out transient CFD simulations to study the influence of turbulence modeling for a BC with a centrally located gas distributor. The authors found that in EE models, the two non-drag forces, namely, the bubble-induced turbulence by Sato and Sekoguchi [13] and turbulent dispersion by Sokolichin et al. [14], play an important role for accurate qualitative and quantitative predictions. Another important finding was the consideration of Reynolds numbers, which varied depending on the dimension (2D or 3D) in which the simulations were carried out. Buwa and Ranade [2] carried out numerical simulations on a 2D rectangular column for a bubble column operating in the heterogeneous regime using 5 different distributor configurations that were centrally located in the bubble column. The authors found that the dynamics of gas–liquid flow depended on the superficial gas velocity, distributor configuration, and liquid phase physical properties on the dynamics of gas–liquid flow.

**Table 1:** Experimental investigation of hydrodynamics in bubble column reactors.

References	Column Size in Volume (m^3^)	Nozzle Size (d, N)	Input Parameters	Measurement Techniques	Parameters Investigated
Azizi et al. [15]	0.007854 **	d = 0.00022–0.00095 m, N = 13–115	U_g_ = 0.01–0.095 m/s	UXCT, RPT	Bubble size, shape, velocity distribution
Liu et al. [5]	0.01875 *	d = NA, N = 18	U_g_ = 0.0008–0.008 m/s	PSTV	Bubble size distribution, bubble dynamics
Juliá et al. [16]	0.012276 *	d = 0.00008 m, N = 137	U_g_ = 0.02–0.1 m/s	LDA, PIV	Bubble-induced turbulence, wake structure
Buwa and Ranade [17]	0.012 *	d = 0.0008 m, N = 8	U_g_ = 0.16–12 m/s	Conductivity probe	Plume oscillation frequency, plume width
Rensen and Roig [4]	0.015075 ***	d = 0.00033 m, N = 14	Q_g_ = 0.000021–0.000054 m^3^/s	Intrusive optical mono-fiber probe	Bubble rise velocity, oscillation, wake
Becker et al. [3]	0.0036 *	d = 0.001 m, N = 24	Q_g_ = 0.0000133 m^3^/s	Optical probes, LDA	Plume oscillation frequency
	0.1709 **	d = 0.0007 m, N = 21 and 89			

Note: * rectangular column, ** cylindrical column, *** square column, d = diameter of holes (m), N = number of holes, U_g_ = superficial gas velocity (m/s), Q_g_ = gas flow rate (m^3^/s). UXCT = Ultrafast X-ray Computed Tomography, RPT = Radioactive Particle Tracking, PSTV = Particle Shadow Tracking Velocimetry, LDA = Laser Doppler Anemometry, PIV = Particle Image Velocimetry, NA = Not applicable.

**Table 2:** Numerical investigation of bubble column reactors.

References	Column Size in Volume (m^3^)	Nozzle Size (d, N)	Input Parameters	Multiphase Models	Drag and Non-Drag Models	Software with Version	Parameter Investigated
Rojas et al. [18]	0.012 *	d = 0.0008 m, N = 8	Q_g_ = 0.000013 m^3^/s	Euler–Euler	Turbulence model, drag, lift, virtual mass coefficient	ANSYS Fluent 19.2	Time-averaged axial liquid velocity, gas holdup, and gas plume oscillation
Fleck and Rzehak [19]	0.011 *	d = NA, N = 8	U_g_ = 0.0014–0.0073 m/s	Euler–Euler	Drag, shear lift, wall lift, turbulent dispersion, virtual mass coefficient	ANSYS CFX 17.2	Bubble plume oscillations
Asad et al. [20]	0.010125 ***	d = 0.001 m, N = 49	U_g_ = 0.0049 m/s	Discrete Bubble Model, Volume of Fluid	Drag, lift, buoyancy	OpenFOAM	Bubble dynamics, flow behavior
Rzehak et al. [21]	0.000155 **	d = NA, N = 19	U_g_ = 0.011–0.032 m/s	Euler–Euler	Drag, shear lift, wall lift, turbulent dispersion, virtual mass coefficient	ANSYS CFX	Bubble size distribution, bubble coalescence, and breakup

Note: * rectangular column, ** cylindrical column, *** square column, d = diameter of holes (m), N = number of holes, U_g_ = superficial gas velocity (m/s), Q_g_ = gas flow rate (m^3^/s), NA = Not applicable.

**Table 3:** Both experimental and numerical investigations of bubble column reactors.

References	Column Size in Volume (m^3^)	Nozzle Size (d, N)	Input Parameters	Measurement Techniques	Multiphase Models	Interfacial Forces	Software (Version)	Parameters Investigated
Sommer et al. [22]	0.003 *	d = 0.0002–0.0006 m, N = 14	U_g_ = 0.002–0.006 m/s	PIV, Shadowgraphy	Euler–Euler	Drag, lift, virtual mass coefficient, wall lubrication, turbulent dispersion	OpenFOAM v10	Bubble size distribution, gas holdup
Tas-Koehler et al. [23]	0.01133 **	NA	U_g_ = 0.0368 m/s	UFXCT	Euler–Euler, PBM	Drag, lift, virtual mass coefficient, wall lubrication, turbulent dispersion	ANSYS CFX 19.2	Void fraction, velocity profiles
Saleh et al. [24]	0.0229 **	d = 0.001 m, N = 37	U_g_ = 0.021 m/s	Electro-resistivity probe	Euler–Euler	Drag, lift, wall lubrication, turbulent dispersion	ANSYS CFX 15.0	Gas holdup, liquid velocity
Al-Naseri et al. [25]	0.02818 **	NA	U_g_ = 0.05–0.45 m/s	RPT, CT	Euler–Euler, PBM	Drag, lift, wall lubrication, turbulent dispersion	ANSYS Fluent 15.0	Local gas holdup, internal effects
Gupta and Roy [26]	0.012 *	d = 0.0008 m, N = 8	U_g_ = 1.33 m/s	RPT	Euler–Euler, PBM	Drag, lift, virtual mass coefficient	ANSYS Fluent 14.0	Compare different drag models and bubble sizes at different velocities
Buwa et al. [27]	0.012 *	d = 0.0008 m, N = 8	U_g_ = 0.0016–0.12 m/s	Wall pressure and voidage fluctuation measurement of [2,17]	Euler–Euler	Drag, lift, virtual mass coefficient	N/A	Effect of superficial gas velocity, H/W ratio, lift force, and numerical diffusion on dynamic and time-averaged flow properties
Buwa and Ranade [28]	0.012 *	d = 0.0008–0.002 m, N = 8	U_g_ = 0.0016–0.14 m/s	Conductivity probe	Euler–Lagragian	N/A	N/A	Plume oscillation frequency, bubble passage frequencies, dimensional analysis
Delnoij et al. [29]	0.01 *	d = 0.0002 m, N = 14	U_g_ = 0.035 m/s	N/A	Euler–Lagragian	Drag, lift, virtual mass coefficient	N/A	Frequency oscillation of the effect of dimensionality, aspect ratio, the effect of momentum transfer due to bubble–bubble collisions

Note: * rectangular column, ** cylindrical column, d = diameter of holes (m), N = number of holes, U_g_ = superficial gas velocity (m/s), RPT = Radioactive Particle Tracking, UFXCT = Ultrafast X-ray Computed Tomography, PBM = Population Balance Model, CT = Gamma-ray-Computed Tomography.

Buwa and Ranade [28], through numerical simulations with a centrally located sparger, found that gas–liquid flow in a bubble column with a centrally located sparger was inherently unsteady, and re-circulatory flow structures in bubble columns were different from gross re-circulations observed in time-average flow measurements. The authors also compared the mixing times predicted by using instantaneous and time-averaged flow fields. It was found that the amount of mixing predicted using time-averaged flow field was much larger than the experimentally observed values, while mixing time predictions based on instantaneous flow fields agreed well with the experimental data. The authors also found that low-frequency oscillations were a function of operating parameters. The plume oscillation periods varied from 5 to 13 s, with the highest time periods for low superficial velocities ranging from 0.0015 to 0.008 m/s. Long et al. [30] found that the wandering motion of bubble plumes can be reproduced by Standard LES (Smagorinsky SGS model), while the k-ε model could only capture the wandering effect at the beginning of the simulation. Some of the limitations of Standard LES (Smagorinsky SGS model) are its inability to resolve bubble-induced large-scale turbulent eddies above the filter scale. Further, the authors found that bubble-induced turbulence had an effect both on the scale of liquid flow with length scales larger and smaller than the bubbles. The authors have proposed a modified Smagorinsky scale model, which incorporates the effect of bubble response on the turbulent eddies in the SGS scale. Takes 20% more time than the k-ε turbulence model.

Rensen and Roig [4] carried out experimental studies to understand the non-stationary behavior of the plumes for a bubble column operating in the heterogeneous regime. The authors observed different flow behavior for low gas flow rates with or without interactions with the walls. Juliá et al. [16] performed experimental studies on a bubble column operating in a homogeneous regime by sparger holes. The authors observed the formation of different zones during the plume formation and bubbles with faster rise velocities during their investigations. Liu et al. [5] carried out experimental studies on the effect of different aspect ratios, fluid properties, and superficial velocities on plume oscillations and bubble size for BCs operating in the turbulent regime. The authors reaffirmed the fact that plume oscillation frequency was a key parameter for mixing and mass transfer in the column, and the dynamics of vortices were a strong function of the aspect ratio. For example, BCs with high aspect ratios have staggered vortices (two opposite counter-rotating vortices on two sides of the bubble plume), while low aspect ratios have the fluid moving up at the center with the plumes and moving downward near the walls (referred to as the Gulf Stream pattern by the authors). Buwa and Ranade [17] carried out numerical studies for superficial velocities of 0.0016 to 0.12 m/s for an aspect ratio of 2.25 to 4.5. The holdup profiles have been found to be a strong function of the method of gas distribution or sparging. For uniformly sparged cylindrical columns, gas holdup profiles were parabolic in nature with zero or finite gas holdup at the column walls. The authors reported that the gas holdup profiles were bell-shaped for higher superficial velocities (>0.03 m/s) but differed from those of low superficial velocities (0.0016–0.015 m/s).

Empirical correlations to obtain gas holdup and liquid velocity have been obtained by various researchers, as shown in Table 4 and Table 5. However, these correlations are always valid across a certain operating range as per the ratio and gas distributor design for the air–water system of bubble columns. The correlations mainly show gas holdup and liquid velocities as a function of superficial velocities, properties of the continuous phase, surface tension, and hole sizes.

**Table 4:** Correlations of gas holdup.

Reference	Correlation
Kumar et al. [31]	$\alpha_{g}=0.728U^{\prime }-0.485U^{\prime 2}+0.0975U^{\prime 3}$ $U^{\prime }=U_{g}\cdot {\left(\rho_{l}^{2}/(\sigma (\rho_{l}-\rho_{g})\cdot g)\right)}^{0.25}\;\;$
Hughmark [32]	$\alpha_{g}={\left(2+\left(\frac{0.35}{U_{g}}\right)\cdot {\left(\frac{\left(\rho_{l}\rho_{g}\right)}{72}\right)}^{\frac{1}{3}}\right)}^{-1}$
Reilly et al. [33]	$\alpha_{g}=0.009+296\;\cdot U_{g}^{0.44}\cdot \rho_{i}^{-0.98}\cdot \sigma^{0.16}\cdot \rho_{g}^{0.19}$
Kawase et al. [34]	$\alpha_{g}=(0.0625\cdot {\left(\rho_{l}\cdot \frac{U_{g}}{\mu_{l}}\right)}^{0.25})/(1-(0.0625\cdot {\left(\rho_{l}\cdot \frac{U_{g}}{\mu_{l}}\right)}^{0.25})$
Hikita et al. [35]	$\alpha_{g}=0.672\cdot f\cdot {\left(U_{g}\cdot \frac{\mu_{l}}{\sigma }\right)}^{0.578}\cdot {\left({(\mu }_{l}^{4}\cdot g)/(\rho_{l}\cdot \sigma^{3})\right)}^{0.131}\cdot {\left(\frac{\rho_{g}}{\rho_{l}}\right)}^{0.262}\cdot {\left(\frac{\mu_{g}}{\mu_{l}}\right)}^{0.107}$ (for pure liquids, f = 1)
Akita and Yoshida [36]	$\frac{\alpha_{g}}{{\left(1-\alpha_{g}\right)}^{4}}=f\cdot {\left(D^{2}\cdot \rho_{l}\cdot \frac{g}{\sigma }\right)}^{\frac{1}{8}}\cdot {\left(g\cdot D^{3}\cdot \frac{\rho_{l}^{2}}{\mu_{l}^{2}}\right)}^{\frac{1}{12}}\cdot \left(\frac{U_{g}}{g\cdot \sqrt{D}}\right)$ (for pure liquids, f = 0.2)
Behkish et al. [37]	$\alpha_{g}=4.94\times 10^{-3}\frac{\left(\rho_{l}^{0.415}\cdot \rho_{g}^{0.177}\right)}{{(\mu }_{l}^{0.174}\cdot \sigma^{0.27})}\cdot U_{g}^{0.553}\cdot \left(\frac{p_{t}}{{(p}_{t}-p_{s})}\;\right)\cdot {\left(\frac{D}{D+1}\right)}^{-0.117}\cdot \Gamma^{0.053}$ $\Gamma =K_{d}\cdot N\cdot d_{o}^{\alpha }$
Şal et al. [38]	$\alpha_{g}=0.2278\cdot {\left(\frac{U_{g}^{2}}{d_{o}\cdot g}\right)}^{0.7767}\cdot {\left(\frac{d_{o}^{3}\cdot \rho_{l}^{2}\cdot g}{\mu_{l}^{2}}\right)}^{0.3649}\cdot {\left(\frac{d_{o}}{D}\right)}^{0.4780}\cdot {\left(\frac{d_{o}^{2}\cdot \rho_{l}\cdot g}{\sigma }\right)}^{0.3961}\cdot {\left(\frac{D^{4}\cdot U_{g}^{2}\cdot \rho_{l}}{d_{o}^{3}\cdot N^{2}\cdot \sigma }\right)}^{0.2402}$
Krishna and Ellenberger [39]	$\alpha_{g}=\alpha_{g,SB}+\alpha_{g,LB}$ $\alpha_{g,LB}=0.268\cdot {\left(U_{g}-U_{df}\right)}^{\frac{4}{5}}/(D^{0.18}\cdot {\left(U_{g}-U_{df}\right)}^{0.22})$ $\alpha_{g,SB}=\alpha_{gm,trans}$

Note: α_g_ = gas holdup (–), *U′* = dimensionless velocity parameter (–), *U_g_* = superficial gas velocity (m/s), $\rho_{l}=liquid\;density$ (m^3^/s), $\rho_{g}=gas\;density$ (m^3^/s), σ=surface tension (N/m), *g* = acceleration of gravity (m/s^2^), *µ_l_* = dynamic viscosity of liquid (Kg/m s), *µ_g_* = dynamic viscosity of gas (Kg/m s), *f* = liquid purity factor (), *D* = column diameter (m), *P_t_* = total pressure (Pa), *P_s_* = saturation pressure (Pa), *K_d_* = empirical constant (–), *N* = number of holes (–), *d_o_* = orifice diameter (m), *Γ* = empirical correlation parameter (–), *α_g,SB_* = gas holdup due to small bubbles (–), *α_g,LB_* = gas holdup due to large bubbles (–), *U_df_* = minimum fluidization gas velocity (m/s), *α_gm,trans_* = transitional gas holdup (–).

**Figure 2:**
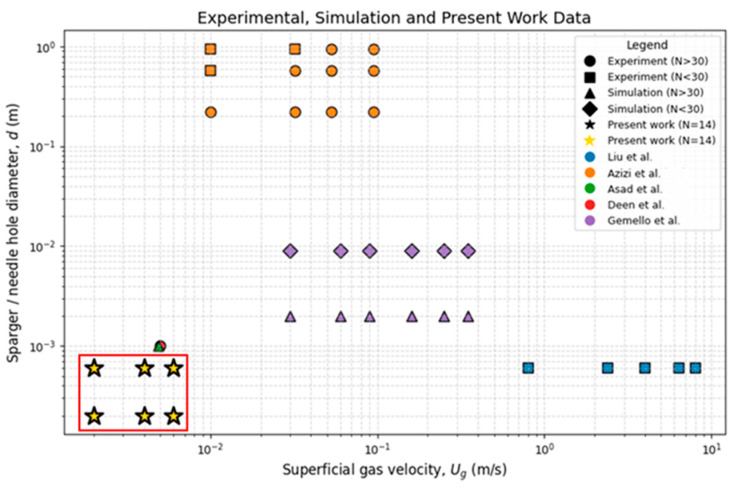
Reported literature data based on the number of holes, hole size, and superficial gas velocity. Symbols represent the number of holes for simulation, experiment, and present work, while authors of the published work represent by Blue color denotes Liu et al. [5], Orange color denotes Azizi et al. [15], Green color denotes Asad et al. [20], Red color denotes Deen et al. [47], Purple color denotes Gemello et al. [48].

Figure 2 depicts the different research works of authors with different sparger designs having different numbers of holes. The most recent works have been chosen since earlier works have been documented well in [40,41]. The figure clearly depicts that no studies are available for hole sizes of 200 µm. Sufficient experimentation and numerical simulations have been carried out for hole sizes greater than 1 mm and for numbers of holes less than 30 or above 30. However, the pitch of the holes has been uniform in most cases.

**Table 5:** Correlations of liquid velocity.

Reference	Correlation
Walter and Blanch [42]	$U_{l}\left(\xi \right)=U_{l,max}\left(1-{\left(\frac{\xi }{\xi_{inv}}\right)}^{2}\right)$ Where $\xi_{inv}=0.7$
Wu et al. [43]	$U_{l}\left(\xi \right)=U_{l,max}\left(1-2.65\cdot \;n^{0.44}\cdot c\xi^{2.65n^{0.44}c}\right)$
Zehner [44]	$U_{l,max}=0.737{\left(U_{g}\cdot D\right)}^{\frac{1}{3}}$
Riquarts [45]	$U_{l,max}={\left(0.21\cdot g\cdot D\right)}^{\frac{1}{2}}\cdot {\left(\frac{U_{g}\cdot \rho_{l}}{g\cdot \mu_{l}}\right)}^{\frac{1}{8}}$
Besagni and Deen [46]	$U_{l}=k\cdot \alpha_{g}^{0.8}\cdot d_{32}^{0.3}\cdot {\left(\rho_{l}/\rho_{g}\right)}^{0.2}\cdot g^{0.1}$

Note: *U_l,max_* = maximum liquid velocity (m/s), *ξ* = dimensionless radial co-ordinates (–), *ξ_inv_* = radial position of flow inversion (–), *U_l_* = superficial liquid velocity (m/s), *n* = empirical constant (–), *c* = empirical constant (–), *k* = empirical constant (–), *U_g_* = superficial gas velocity (m/s), *D* = column diameter (m), *g* = acceleration of gravity (m/s^2^), $\rho_{l}=liquid\;density$ (m^3^/s), $\rho_{g}=gas\;density$ (m^3^/s), *µL* = dynamic viscosity of liquid (Kg/m s), *d*_32_ = sauter mean diameter of bubble (m), *α_g_* = gas holdup (–), σ=surface tension (N/m).

In the previous paragraphs, experimental and numerical studies carried out by the researchers over the past three decades focusing on plume dynamics, the significance of different turbulence models (like the k-ε and LES models), and the role of different force models (drag, lift, etc.) used in Eulerian–Eulerian simulations to predict important parameters like the liquid velocity and holdup profiles in BCs have been presented. Biochemical and environmental processes depend on microorganisms and need to be operated at low superficial velocities. The literature review shows that most experimental and numerical works in rectangular bubble columns have a centrally located distributor. The studies have concentrated on numerical and experimental studies on meandering plumes for superficial velocities in the turbulent regime. Studies on hydrodynamics in three-dimensional (3D) BCs using uniform distributors and low superficial velocities and understanding the flow structures in the homogeneous regime are limited. These characteristics play an important role in bio-reactions and wastewater processes that might operate in the homogeneous regime and require the understanding of flow structures and turbulence quantities inside contacting equipment, especially with respect to the longevity of the microorganisms. This depends on the importance of the distributor design (hole size and pitch), geometry (like aspect ratio), and operating parameters (like the superficial gas velocity). Numerical studies in 3D bubble columns focused on hydrodynamics (including the flow structures and hold up, velocity, and turbulence profiles), which depend on the multiphase and turbulence models chosen. While experimental studies have used conductivity probes for gas holdup measurement and PIV for vertical structures and axial velocity profiles. In an extremely interesting research study by Sommer et al. [22], the focus lay in measuring and predicting (using CFD simulations) radial profiles of liquid velocities and holdup characteristics. However, the investigations were limited to quantitative studies and not the transient flow structures, both in terms of velocities and holdup. Further, the quantitative profiles were mostly focused on an axial location near the top of the column, and no turbulent quantities like the turbulent kinetic energy (TKE) were investigated. The simulations were carried out in only a quarter of the geometry instead of the full geometry.

The present work is focused on carrying out full-scale 3D CFD simulations for a lab-scale rectangular bubble column (0.5 m height, 0.1 m width, 0.05 m depth) of aspect ratio 5, a uniform distributor plate with hole sizes in the range of 200–600 µm, and superficial velocities less than 0.01 m/s, Sommer et al. [22] using the EE approach and the k-ε turbulence model. The objectives of the present work are (1) to qualitatively understand the transient gas flow dynamics originating from the chosen distributor design for two different hole sizes and three superficial velocities; (2) to quantitatively analyze the radial velocities, gas holdup, and TKE profiles for three different axial positions in the column; and (3) to develop empirical correlations for gas holdup and liquid velocities and compare them with available correlations in the literature. Due to the transient nature and low velocity of flow, we deliberately select the simplest turbulence model (the standard k-ε turbulence model) in the current study that would form a strong basis for future studies with higher-order models.

## 2. Mathematical Details

This section discusses the governing equations for a two-phase system and the correlations for drag and non-drag forces used for the simulation studies.

### 2.1. Geometric and Mesh Details

This section presents a schematic of the computational geometry used in the study, along with the hexahedral mesh employed for the simulation.

**Figure 3:**
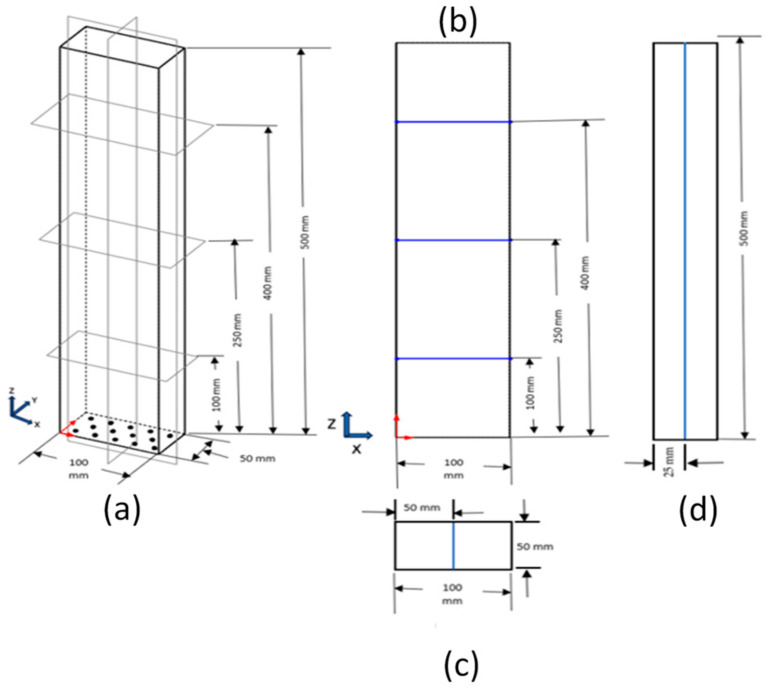
Schematic of the geometry depicting appropriate dimensions, hole positions, vertical planes in X and Y directions, and horizontal lines for profiles (**a**) 3D view, (**b**) 2D view of central XZ plane. The three different horizontal locations are shown by dark blue color, (**c**) 2D view of central XY plane. Center line is shown by light blue color, and (**d**) 2D view of central YZ plane. Center line is shown by light blue color.

**Figure 4:**
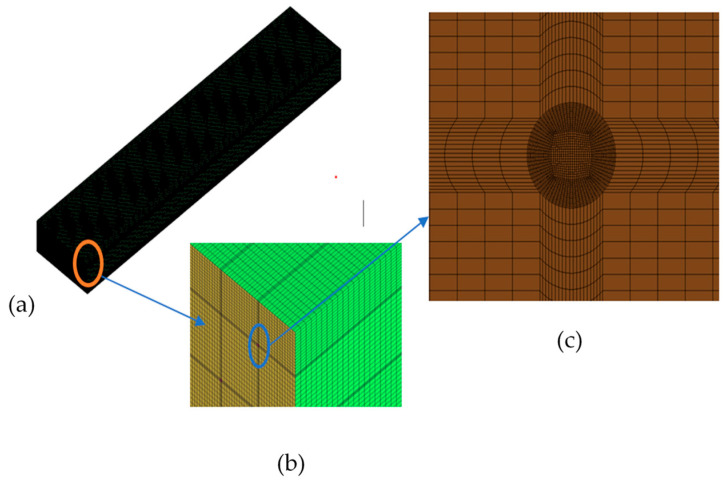
Mesh details: (**a**) 3D mesh of entire geometry, (**b**) zoomed view of uppermost corner of the mesh represented by orange circle and blue arrow, (**c**) zoomed view of the inlet hole represented by blue circle and blue arrow.

Figure 3a shows the 3D view of the rectangular bubble column considered for study. The typical positions of the YZ planes and the XZ planes are shown. These are shown in forthcoming figures where velocity vectors and volume fraction contours are shown for the XZ plane at Y = 0.025 m and the YZ plane at a particular time and X varying from 0.01 to 0.1 m at an interval of 0.01 m. Further, the radial profiles of gas volume fractions, liquid velocity, and turbulent kinetic energy have been taken at 3 axial positions depicted in Figure 3b. Figure 4a shows the full 3D mesh of the rectangular bubble column, while Figure 4b,c are the zoomed views to show the hexahedral meshes of the corner and inlet hole of the geometry.

### 2.2. Boundary Conditions

No-slip boundary conditions have been used at all the walls. At the outlet, we make an assumption that the gas bubbles escape the domain with a terminal rise velocity. The height of the liquid was set equal to 0.5 m in all the simulations. Inlet values were considered as the inlet velocity of the gas.

### 2.3. Equation for Mass, Momentum, and Turbulence for Each Phase

(1) $$\frac{\partial }{\partial t}\left(\alpha_{G}\rho_{G}\right)+\nabla \cdot \left(\alpha_{G}\rho_{G}u_{G}\right)=0$$

$$\frac{\partial }{\partial t}\left(\alpha_{L}\rho_{L}\right)+\nabla \cdot \left(\alpha_{L}\rho_{L}u_{L}\right)=0$$

(2) $$\frac{\partial }{\partial t}\left(\alpha_{G}\rho_{G}u_{G}\right)+\nabla \cdot \;\left(\alpha_{G}\rho_{G}u_{G}\;⊗\;u_{G}\right)=-\alpha_{G}\nabla p+\nabla \cdot \left(\alpha_{G}T_{G}\right)+{(\alpha }_{G}\rho_{G}g)+F_{G}^{inter}$$

(3) $$\frac{\partial }{\partial t}\left(\alpha_{L}\rho_{L}u_{L}\right)+\nabla \cdot \;\left(\alpha_{L}\rho_{L}u_{L}\;⊗\;u_{L}\right)=-\alpha_{L}\nabla p+\nabla \left(\alpha_{L}T_{L}\right)+{(\alpha }_{L}\rho_{L}g)+F_{L}^{inter}$$

where Equations (1)–(3) denotes mass and momentum equations for each phase. *F^inter^* is the interfacial momentum exchange force, the subscripts *G* and *L* denote the gas and liquid phase, respectively, and *α*, *ρ*, *p*, *g*, and *u* denote the volumetric phase fraction, density, pressure, acceleration of gravity, and phase velocity, respectively.

The stress tensor *T* (in Equation (4)), including molecular and turbulent stresses, is defined as

(4) $$T=\mu^{eff}\left({\left(\nabla u+(\nabla u\right)}^{T})-\frac{2}{3}\;({\nabla \cdot u}_{k})I\right)$$

where *μ^eff^* represent the effective viscosity while *I* is a 3 × 3 identity matrix.

(5) $$\mu^{eff}=\mu^{mol}+\mu^{turb}$$

where *μ^mol^* and *μ^turb^* are the molecular and turbulent eddy viscosity, respectively as shown in Equation (5).

Turbulence in the liquid phase was modeled using the standard k–ε model (as given below in Equations (6) and (7)),

(6) $$\frac{\partial }{\partial t}\left(\alpha_{L}\rho_{L}k_{L}\right)+\nabla \cdot \left(\alpha_{L}\left(\rho_{L}U_{L}k_{L}-\left(\mu_{L}+\frac{\mu_{t,L}}{\sigma_{k}}\right)\;\nabla k_{L}\right)\right)=\alpha_{L}\left(P_{L}-\rho_{L}\varepsilon_{L}\right)+T_{LG,\;k}$$

(7) $$\frac{\partial }{\partial t}\left(\alpha_{L}\rho_{L}k_{L}\right)+\nabla \cdot \left(\alpha_{L}\rho_{L}U_{L}\varepsilon_{L}-\left(\mu_{L}+\frac{\mu_{t,L}}{\sigma_{\varepsilon }}\right)\;\nabla \varepsilon_{L}\right)=\alpha_{L}\frac{\varepsilon_{L}}{k_{L}}\left(C_{1}P_{L}-C_{1}\rho_{L}\varepsilon_{L}\right)+T_{LG,\;\varepsilon }$$

where $P_{L}$ is the turbulence production due to shear. The constants $C_{1},\;C_{2},\;\rho_{k}\;and\;\rho_{\varepsilon }\;$ take the standard values of 1.44, 1.92, 1.0, and 1.3, respectively. The terms involving T_LG_ are the sources and sinks of turbulence due to the presence of the bubbles.

The liquid phase turbulent viscosity ($\mu_{t,L}$) is modeled as given below in Equation (8):

(8) $$\mu_{t,L}=\;C_{\mu }\rho_{L}\;\left(\frac{k_{L}^{2}}{\varepsilon_{L}}\right)$$

The gas phase turbulent viscosity is determined from the following in Equation (9):

(9) $$\mu_{t,G}=\;\frac{\rho_{G}}{\rho_{L}}\left(\mu_{t,L}\right)$$

### 2.4. Models for Drag and Non-Drag Forces

Bubble diameter in the present work was specified based on the bubble sizes provided by Sommer et al. [22] for the validation of the model. Further, the turbulence levels are low, and the contribution of turbulent eddies larger than the bubble size on bubble dispersion is rather negligible. Therefore, simulations without considering turbulent dispersion are closer to the experimental results than those obtained with artificially high dispersion of bubbles generated by considering the turbulent dispersion term. The qualitative snapshots of simulated plume oscillations were not sensitive to the virtual mass force term. It was observed that the effect of the turbulent dispersion force was less significant for the cases studied in the present work. However, Mudde and Van Den Akker [7] have shown the dependence of plume oscillation on the virtual mass term and lift force terms. Hence, both have been included during the EE simulations in the present work.

The term $F^{inter}$ represents the sum of all forces between the phases are given by Equations (10) and (11):

(10) $$F_{G}^{inter}=\;-\;F_{L}^{inter}=\;\sum_{i}F^{i}$$

(11) $$\sum_{i}F^{i}=F^{drag}+F^{lift}+F^{wall}+F^{disp}+F^{vm}.$$

#### 2.4.1. Drag Force

In the present work, the correlation of Ishii and Zuber [49] has been used as given by Equation (12) below:

(12) $$F^{drag}=\;K_{GL}\;(u_{l}-\;u_{g})$$

where the interphase momentum exchange coefficient *K_GL_* is given by Equation (13)

(13) $$K_{GL}=\frac{3}{4}\;\frac{C_{D}\alpha_{g}\rho_{l}\left|u_{rel}\right|}{d_{b}}$$

Here *u_rel_* = *u_l_* − *u_g_* is the relative velocity between the liquid and gas phases, *d_b_* is the bubble diameter, and *C_D_* is the drag coefficient given by Equation (14).

(14) $$C_{D}=\left[max\left[minC_{D,\;sphere},min\left[C_{D,\;ellipse},C_{D,\;cap}\right]\right]\right]$$

where all the drag coefficients in the bracket are given by Equation (15) below

(15) $$C_{D,\;sphere}=\frac{24}{{Re}_{B}}\;\left(1+0.1\;{Re}_{B}^{0.75}\right);\;C_{D,\;ellipse}=\frac{2}{3}\;\sqrt{Eo}\mathrm{and}\;C_{D,\;cap}=\frac{8}{3};\;{Re}_{B}=\left|u_{rel}\right|d_{b}v_{L}^{-1};Eo=g∆\rho d_{B}^{2}\sigma^{-1}$$

#### 2.4.2. Lift Force

In the present work, the lift force was used (Equation (16)) as follows:

(16) $$F^{lift}=\;{-∁}_{L}\rho_{L}\alpha_{G}\;\left|u_{G}-u_{L}\right|\;\times rot\;{(u}_{L})$$

The lift coefficient was calculated using the empirical model in Equation (17), as proposed by Tomiyama et al. [50]

(17) $$C_{L}=\left\{\begin{array}{c} \min\left[0.288\tanh\left(0.121Re\right),f\left(Eo\right)\right],\;Eo<4\\ f\left(Eo\right),\;4\;\leq \;Eo\;\leq \;10.7 \end{array}\right.$$

$$f\left(Eo\right)=0.00105{Eo}^{3}-0.0159{Eo}^{2}-0.0204Eo+0.474.$$

#### 2.4.3. Wall Lubrication Force

In the present work, the wall lubrication force was used (Equation (18)) as below:

(18) $$F^{wall}=\;\frac{2}{d_{B}}C_{W}\rho_{L}\alpha_{G}{\left|u_{G}-u_{L}\right|}^{2}\;\hat{y}\;$$

where ${\left|u_{G}-u_{L}\right|}^{2}$ is the wall tangential component of the relative velocity between phases and $\hat{y}$ is the unit vector normal to the wall pointing into the fluid. The coefficient *C_W_* is calculated by Equations (19) and (20) as formulated by Hosokawa et al. [51]

(19) $$C_{w}\left(y\right)=f\left(Eo\right){\left(\frac{d_{B}}{2y}\right)}^{2}$$

(20) $$f\;\left(Eo\right)=0.0217\;Eo$$

where *y* is the distance between the bubble and the wall.

#### 2.4.4. Turbulent Dispersion Force

The dispersion of bubbles due to liquid velocity fluctuation is described by the turbulent dispersion force. In the present work, the model of Burns et al. [52] was used and is given by Equation (21) below:

(21) $$F^{disp}=\;-\frac{3}{4}\;C_{D}\frac{\alpha_{G}}{d_{B}}\left|u_{G}-u_{L}\right|\frac{\mu_{L}^{turb}}{\sigma_{TD}}\;\left(\frac{1}{\alpha_{L}}+\frac{1}{\alpha_{G}}\;\right)\nabla \alpha_{G}$$

where $\sigma_{TD}$ is the turbulent Schmidt number and is set to 0.9.

#### 2.4.5. Virtual Mass Force

The virtual mass force describes that a portion of the liquid mass is accelerated due to the flow acceleration and velocity difference between the phases. In the present work, the following Equations (22) and (23) was used for incorporating the virtual mass force [53]:

(22) $$F^{vm}=\;\rho_{L}\alpha_{G}C_{vm}\frac{D_{L}u_{L}}{Dt}-\;\frac{D_{G}u_{G}}{Dt}$$

where the substantial derivative of a scalar or vector field *ϕ* is defined

(23) $$\frac{D_{i}\emptyset }{Dt}=\frac{\partial \emptyset }{\partial t}+\left(u_{i}\;\nabla \right)\;\emptyset$$

and the virtual mass coefficient $C_{vm}$ is set to 0.5.

### 2.5. Grid Sensitivity

**Figure 5:**
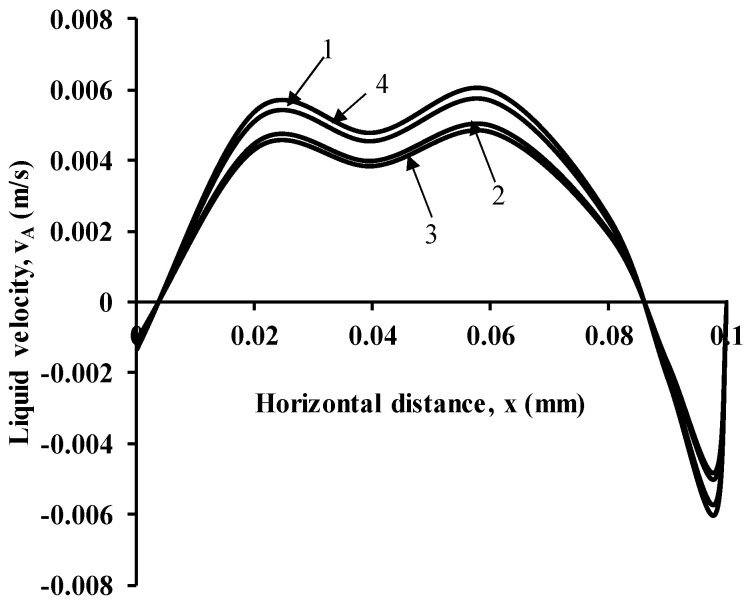
Grid sensitivity of different meshes for radial velocity profile at an axial location z = 0.25 m for sparger hole diameter of 200 µm and superficial gas velocity of 0.002 m/s 1. Coarse grid 2. Medium 3. Medium 4. Fine grid. *X* axis range for distance is from 0 to 0.1.

The grid sensitivity is shown in Figure 5. Four different grids have been chosen for grid sensitivity, comprising a coarse (0.55 million), medium (1 million and 1.35 million), and a fine grid (2 million). The medium grids are divided into two, namely medium1 (1 million) and medium2 (1.35 million) grids. It was observed that as the grid size increased, the agreement between the subsequent grids of long-time-averaged axial velocity decreased and then increased. The time-averaged radial velocity with two medium grids was within 3%, and between coarse and medium grids was within 15%, while that of fine and two medium grids was within 20%. The medium grid results were also consistent with the experimental data of Sommer et al. [22].

## 3. Results and Discussion

In the present section, first, the model is validated with the experimental data of Sommer et al. [22]. The qualitative patterns in terms of volume fraction contours and liquid velocity vectors for two hole sizes (200 and 600 µm) and the three superficial velocities (0.002, 0.004, and 0.006 m/s) mentioned earlier are presented. The quantitative patterns in terms of gas volume fraction profiles, liquid velocities, and turbulent kinetic energy are presented.

### 3.1. Model Validation

**Figure 6:**
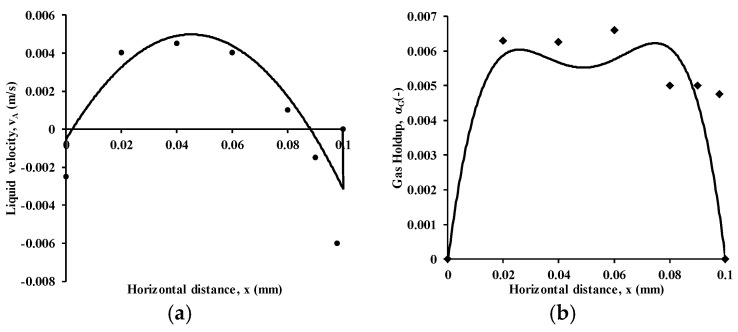
Comparison of predicted radial profiles of liquid velocities and gas holdup with experimental data from the literature, Sommer et al. [22] for a superficial gas velocity of 0.002 m/s and sparger hole diameter of 200 µm at two axial locations z = 0.25 m (**a**) liquid velocity (**b**) gas holdup. Filled round and rhombus symbols denote experimental data while lines denote simulations. *X* axis range for distance is from 0 to 0.1.

The CFD model is validated using the experimental data of Sommer et al. [22]. The model predictions matched well with a maximum deviation of ~8%, as can be observed in Figure 6. Time-averaged radial velocity and gas holdup profiles at an axial location of z = 0.25 m have been chosen for comparison of model predictions. The superficial gas velocity has been considered as 0.002 m/s, and the sparger hole diameter is 200 µm. The deviations in liquid velocities are ±10%, while those of gas holdup are ±13%. The deviations are attributed to the use of the standard k-ε turbulence model, and a higher-order turbulence model would be considered in future studies.

### 3.2. Effect of Drag and Non-Drag Models on Hydrodynamics: Sensitivity Analysis

A sensitivity analysis to observe the effect of drag and non-drag models on the predictions of liquid axial velocity profiles was carried out for the hole sizes and superficial gas velocities considered in the study. Due to an increase in the overall number of figures, the plots of the mentioned sensitivity study are not provided. For some velocities and hole sizes, like all velocities for 600 µm hole sizes, only the DL model was sufficient for time-averaged axial liquid velocities and gas holdup profiles.

However, for 0.002 m/s and 200 µm hole size, the instantaneous profiles depended largely on the inclusion of wall lubrication and turbulent dispersion forces. Transient flow patterns depicted that inclusion of only DL predicted liquid velocity profiles that were varyingly different, with a central peak occurring at all three axial locations, while inclusion of the WL model showed the peak nearer to the wall for intermediate times between 100 s and 300 s. Similarly, inclusion of turbulent dispersion improved the prediction of both liquid velocity and gas holdup profiles. Time-averaged velocities also matched well with experimental data when DL-WL-TD was used. TKE profiles were also predicted for all simulations with DL models, except for 200 µm hole size and 0.002 m/s superficial velocity, where DL-WL-TD was used, and for 0.006 m/s, where DL-WL-TD-VM was used.

The importance of the turbulent dispersion force was also tested by comparing the DL model with the DL-TD model. Its impact was found to be particularly important while investigating the effect of time-averaged velocity for a 600 µm hole size and 0.004 m/s superficial velocity, where the DL model showed a peak in the central region, while DL + TD showed a slightly offset peak, denoting that the velocity profile is dependent on the turbulent dispersion force for predicting the effect of liquid eddies. However, due to the use of the k–ε model, the actual magnitude deviates from the anticipated experimental data, as shown later in Section 3.3.

Bubble Reynolds numbers are <50; therefore, the Ishii–Zuber [49] correlation performed better than the Schiller–Naumann [11] correlation in all cases. It was predominantly tested for cases where the DL model was used. The most important influence on the bubble movement in the Euler–Euler (EE) framework depends on the drag and lift forces. The drag force depicts the force experienced by the gas (dispersed phase) due to the liquid (continuous phase), while the lift force depicts the force experienced by the gas due to the surrounding pressure and stress at the surface of bubbles. As per the Reynolds number range (Re ≤ 50) and Eötvös number (Eo) range, the Ishii and Zuber [49] and Naumann and Schiller [11] were compared. Predictions provided by the former matched better with experimental data in terms of gas holdup profiles and liquid–gas velocities. Hence, the Ishii and Zuber [49] model was chosen. The lift force was calculated using the lift coefficient formulated by the Tomiyama model [54], which was also found to be the best for CFD predictions by Masood and Delgado [10]. Virtual mass coefficient was taken to be 0.5, while the turbulent dispersion force of Burns et al. [52] and the wall lubrication force by Hosokawa et al. [51] was found to be the best virtual mass force that has been seen to have a significant impact on the gas–liquid flows in terms of its complexity.

### 3.3. Qualitative Analysis: Effect of Sparger Hole Size on Transient Flow Patterns During Gas Plume Development

Though the superficial velocities chosen for the present work suggest a homogeneous regime, the bubble movement is anticipated to be different due to the use of a different distributor (with a different pitch and hole size than conventional distributors). Hence, in this section, the effect of superficial gas velocity on transient gas volume fractions and the axial velocity in the form of velocity vectors for two different hole sizes, 600 µm and 200 µm, has been presented.

#### 3.3.1. Volume Fraction Contours and Velocity Vectors for 600 µm Inlet Diameter

**Figure 7:**
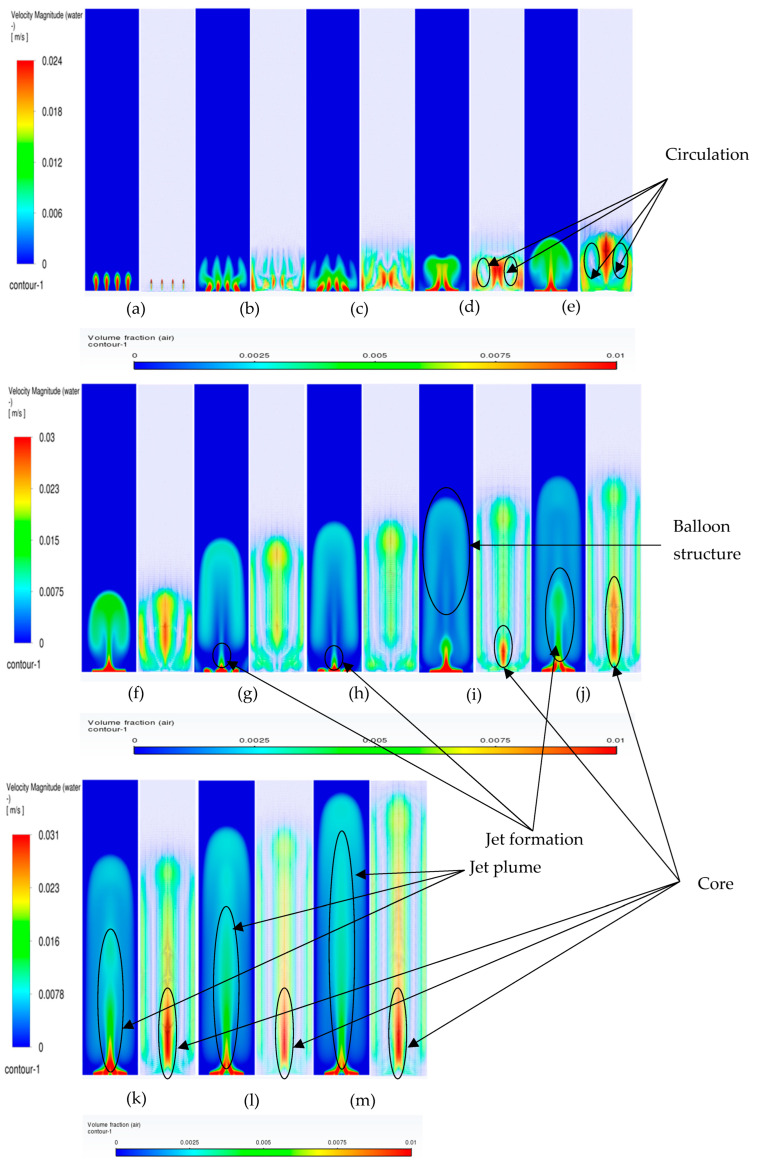
Instantaneous volume fractions and velocity vectors depicting flow structures in the gas–liquid bubble column with a hole diameter of 600 µm and superficial gas velocity of 0.002 m/s for the central plane at X = 0.025 m. (**a**) t = 5 s (**b**) t = 10 s (**c**) t = 15 s (**d**) t = 20 s (**e**) t = 25 s (**f**) t = 30 s (**g**) t = 40 s (**h**) t = 45 s (**i**) t = 50 s (**j**) t = 55 s (**k**) t = 60 s (**l**) t = 65 s (**m**) t = 75 s.

In this section, the transient flow patterns are analyzed during different phases of bubble plume formation and corresponding recirculations (vertical structures) of the liquid velocity near the plume. The current discussion is limited to the central plane in the XZ plane, as shown in Figure 3b. Figure 7 depicts the gas phase volume fraction and velocity vectors for a superficial gas velocity of 0.002 m/s. Initially, during the first 20 s (also denoted in several places as ‘s’) (Figure 7b), it is observed that the gas from individual holes rises for a few millimeters in water, causing tiny jets of gas into water, and corresponding vertical structures develop. However, at 25 s (Figure 7c), the jets start deviating from their straight path and merging with each other, creating a certain pattern that we term the bull-horn pattern. At t = 30 s (Figure 7d), a single jet with high velocity travels a certain distance and forms a cap-shaped structure. The cap-shaped structure of the gas causes a recirculatory structure in the surrounding liquid, with the jet region in the middle with the highest velocity, a region of upper velocity, and a downward velocity near the wall. Two circulation cells are seen between the upward (also referred to as vertical structures) liquid velocity zone and the lower liquid velocity zone, having an inclination with the lower end towards the center and upper end towards the wall, as shown in Figure 7e. These circulation cells are different in size from the usually developed circulation cells observed in the bubble columns operating in the homogeneous regime investigated by the authors in the literature. The two circulation cells are similar to the ones reported in meandering plumes [1,2] and can be interpreted as cells trapped between the upward and downward velocities. As time progresses, the cap detaches with an elongation and moves upward. At this point, the flow patterns are similar to bubbly flows from a centrally located single-holed orifice. The circulation cells retain their size and position observed during 40 s (Figure 7f). After t = 55 s (Figure 7i), the cap-shaped structure does not detach and keeps on elongating and transforms into a balloon structure at t = 70 s (Figure 7l), similar to that observed in the jetting regime in reported literature. The velocity vectors representing circulation cells increase in size with the passage of time (increase in height and decrease in thickness) as observed at 75 s (Figure 7m). A steady state was observed when simulations were carried out after 150 s. It should also be noted that the velocity magnitudes of the plume jet and the upward and lower velocities decrease during the last four instantaneous time sequences. The instantaneous velocities are higher than the time-averaged velocities as reported by Buwa and Ranade [2]. The anticipated reason may be due to the decrease in air bubbles and hence the liquid recirculation as the column reaches steady state.

**Figure 8:**
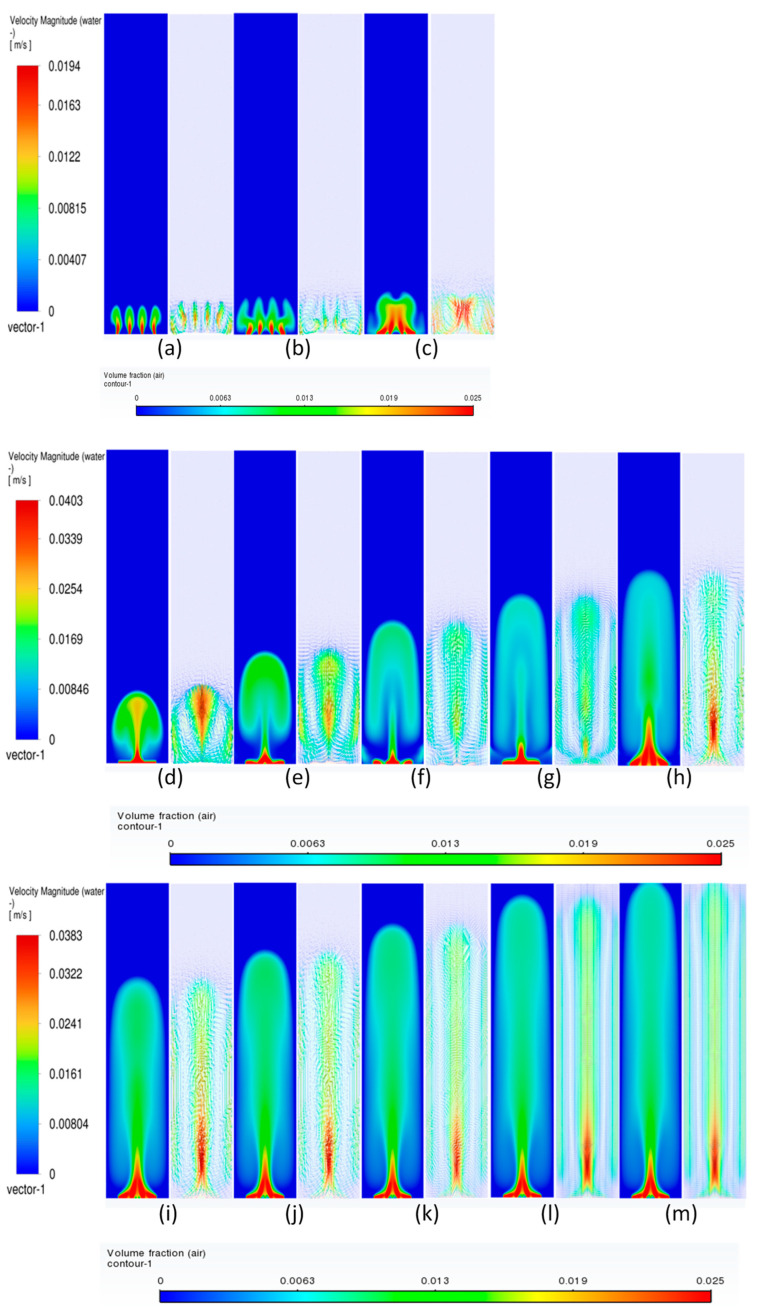
Instantaneous volume fractions and velocity vectors depicting flow structures in the gas–liquid bubble column with a hole diameter of 600 µm and superficial gas velocity of 0.004 m/s for the central plane at X = 0.025 m. (**a**) t = 5 s (**b**) t = 10 s (**c**) t = 15 s (**d**) t = 20 s (**e**) t = 25 s (**f**) t = 30 s (**g**) t = 35 s (**h**) t = 40 s (**i**) t = 45 s (**j**) t = 50 s (**k**) t = 55 s (**l**) t = 60 s (**m**) t = 63 s.

Transient simulations were carried out for superficial velocities of 0.004 m/s. A similar but faster sequence occurs in flow patterns. Figure 8 shows the details of the process. Further, the detachment and merging of the cap-shaped structure are seen in a different form. The bullhorn pattern is replaced by a larger, inverted cup-shaped structure. The structures after 30 s in the present case differ from the ones in Figure 7 in that the jet is always encompassed in the cap-based structure. Further, the balloon-shaped structure is an integrated part of the jet as compared to the lower superficial velocities, where the jet and the balloon structure are decoupled from each other. The cap-shaped structure may be thought of as the outer layer, which encompasses the inner layer (or core of the overall structure), consisting of the balloon structure. The volume fraction magnitudes in the cap-shaped structures are lower than 0.006, while the ones in the balloon structure are in the range of 0.015–0.02. Similarly, the velocity patterns show multiple layers in the balloon-shaped structures, with the innermost layer similar to the shape of a jet plume (like a candle flame) having the highest velocity magnitude. Figure 8e–h shows the development of the overall plume where the core of the plume/flame (denoted by the red color) develops. The flame structure is developed at t = 50 s and maintains approximately the same position till the end of the time simulated. However, Figure 8i–l shows only elongation of the balloon-shaped structure with little increase in the width and height. The circulation cells, however, have the lowest and most consistent spatio-temporal magnitudes and keep increasing in the vertical direction. The inclined nature of the circulation cells is observed in Figure 8e–h. The velocity magnitudes near the wall are similar in Figure 8e–h. An observation similar to the ones in Figure 7 can be found.

**Figure 9:**
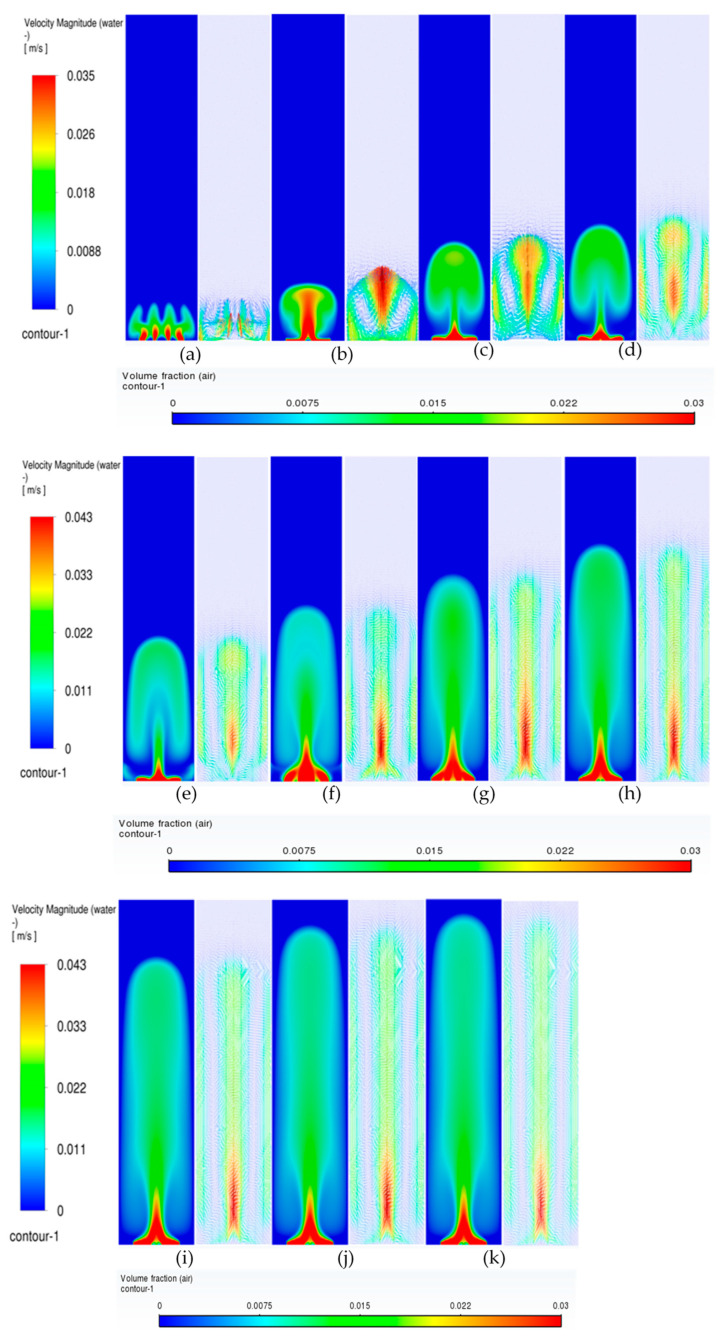
Instantaneous volume fractions and velocity vectors depicting flow structures in the gas–liquid bubble column with a hole diameter of 600 µm and a superficial gas velocity of 0.006 m/s for the central plane at Y = 0.025 m. (**a**) t = 5 s (**b**) t = 10 s (**c**) t = 15 s (**d**) t = 20 s (**e**) t = 25 s (**f**) t = 30 s (**g**) t = 35 s (**h**) t = 40 s (**i**) t = 45 s (**j**) t = 50 s (**k**) t = 53 s.

When the superficial velocities increase to 0.006 m/s, a similar sequence as for the previous superficial gas velocity is observed. Figure 9 shows the details of the process. The structures after 30 s in the present case differ from the ones in Figure 7 in that the jet is always encompassed in the cap-based structure. Further, the balloon-shaped structure is an integrated part of the jet as compared to the lower superficial velocities, where the jet and the balloon structure are decoupled from each other.

**Figure 10:**
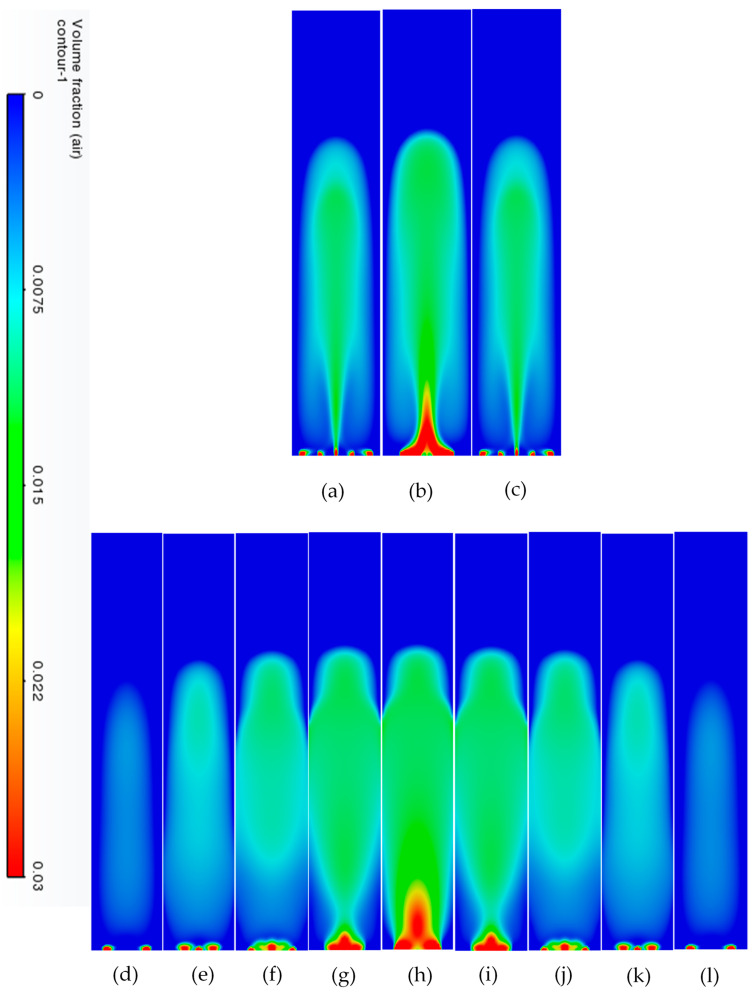
Spatial variation in volume fraction at t = 40 s for 600 µm and superficial gas velocity of 0.006 m/s. (**a**) Y = 0.01 m (**b**) Y = 0.025 m (**c**) Y = 0.04 m (**d**) X = 0.01 m (**e**) X = 0.02 m (**f**–**l**) at every 0.01 m till 0.09 m.

Figure 10 shows the different volume fractions due to the hole structure at a particular time instant. These snapshots help us identify some characteristics of spatial variation in gas at a specific time instant. One of the very clear observations is the symmetric nature (in both YZ and XZ planes) of the flow at the superficial gas velocity of 0.002 m/s.

#### 3.3.2. Volume Fraction Contours and Velocity Vectors for 200 µm Inlet Diameter

**Figure 11:**
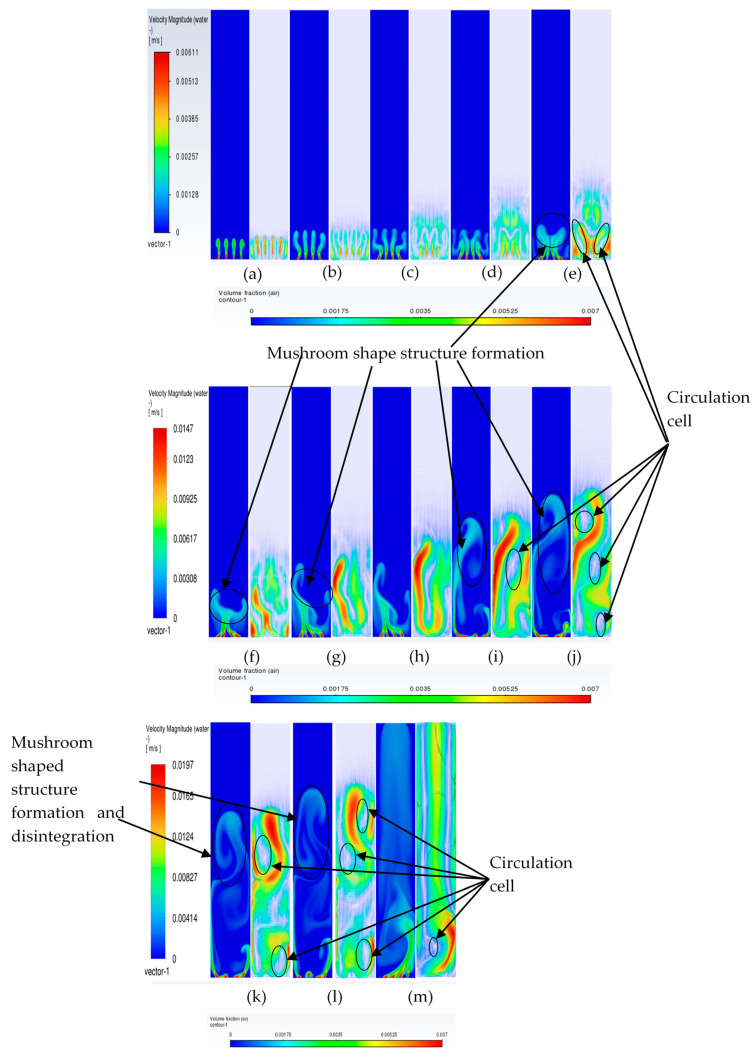
Instantaneous volume fractions and velocity vectors depicting flow structures in the gas–liquid bubble column with a hole diameter of 200 µm and a superficial gas velocity of 0.002 m/s for the central plane at Y = 0.025 m. (**a**) t = 10 s (**b**) t = 20 s (**c**) t = 30 s (**d**) t = 40 s (**e**) t = 50 s (**f**) t = 60 s (**g**) t = 70 s (**h**) t = 80 s (**i**) t = 90 s (**j**) t = 100 s (**k**) t = 110 s (**l**) t = 120 s (**m**) t = 130 s.

To understand the transient re-circulatory flow structures in the rectangular bubble column for a 200 µm inlet diameter, transient simulations have been carried out for all the superficial velocities used in the study. Interesting flow patterns at different timescales are observed both for the flow of gas and the liquid. Figure 11 shows the transient dynamics of the gas plume in terms of the volume fractions and velocity vectors for the central vertical plane at a depth of 0.025 m. The volume fractions indicate that in the early time period (<20 s), the gas rises from the inlets as individual jets, which start merging, and by the end of 40 s form a bullhorn structure as shown in Figure 11d. This structure develops till 60 s (Figure 11f), and with further progress in time, a plume develops, which has a drift to the left wall with the initiation of a mushroom-shaped structure at the tip at t = 70 s (Figure 11g). The mushroom shape can be distinctly observed at t = 90 s (Figure 11i). A similar structure is observed during many other processes, an example being the coalescence of droplets [55]. The structure develops and moves towards the right wall up to t = 100 s. At t = 110 s, it hits the right wall, after which it breaks into two parts. One part disintegrates and moves down while the other part develops another mushroom structure. However, after the next 10 s, a stagnant plume is observed. The velocity vectors show that during the formation of the first mushroom shape, the recirculation is created with downward velocities of the liquid in the right section of the column, in the lowermost part of the column. An inclined recirculation cell can be seen during this period, as can be observed at t = 90 s (Figure 11i). However, in 10 s, multiple circulation cells (3 cells as can be observed) are retained at t = 110 s (Figure 11k), and two cells bigger in size and higher circulation strength can be observed for t = 120 s (Figure 11l). The meandering plume (in the form of a mushroom shape) also causes mixing with circulation cells on the left and right of the central plume.

**Figure 12:**
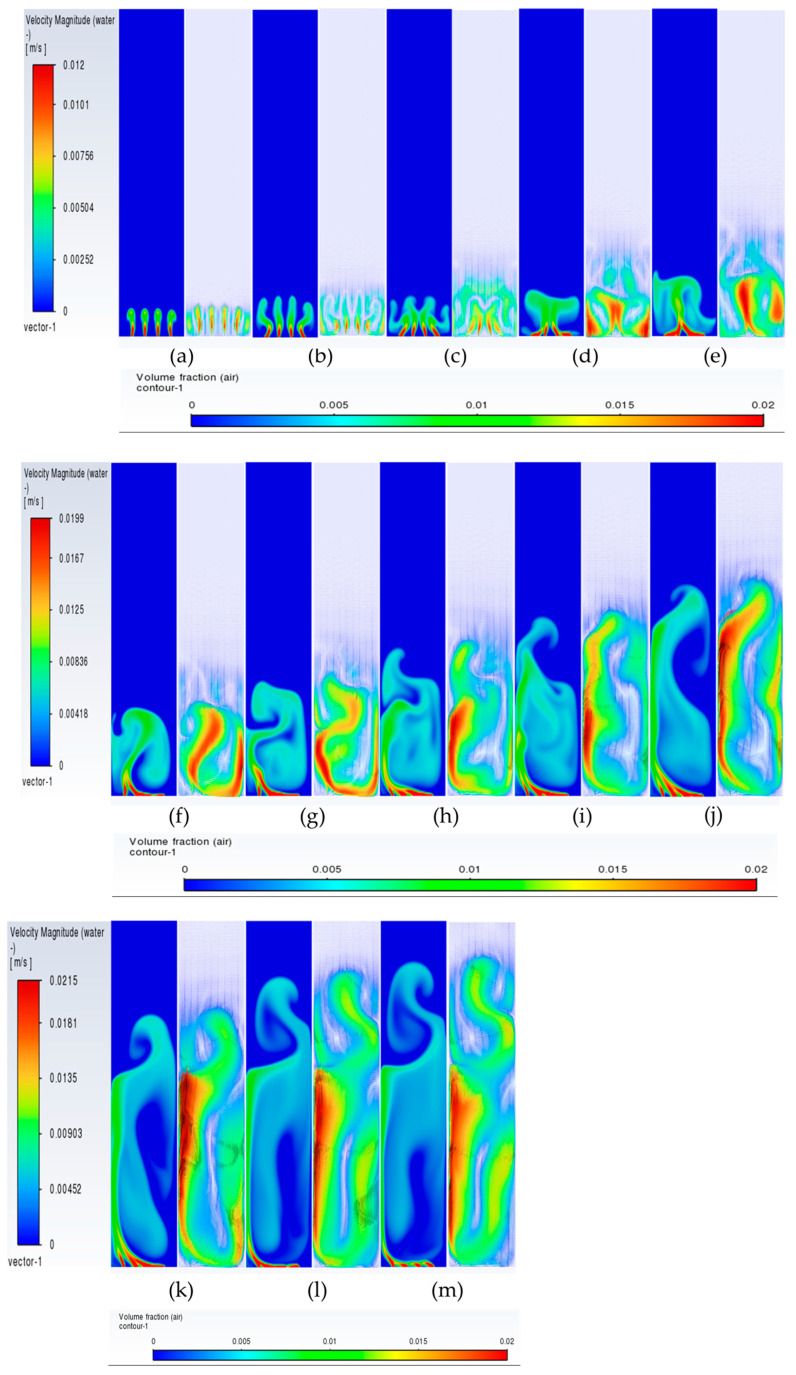
Instantaneous volume fractions and velocity vectors depicting flow structures in the gas–liquid bubble column with a hole diameter of 200 µm and a superficial gas velocity of 0.004 m/s for the central plane at Y = 0.025 m. (**a**) t = 10 s (**b**) t = 20 s (**c**) t = 30 s (**d**) t = 40 s (**e**) t = 50 s (**f**) t = 60 s (**g**) t = 70 s (**h**) t = 80 s (**i**) t = 90 s (**j**) t = 100 s (**k**) t = 110 s (**l**) t = 120 s (**m**) t = 125 s.

**Figure 13:**
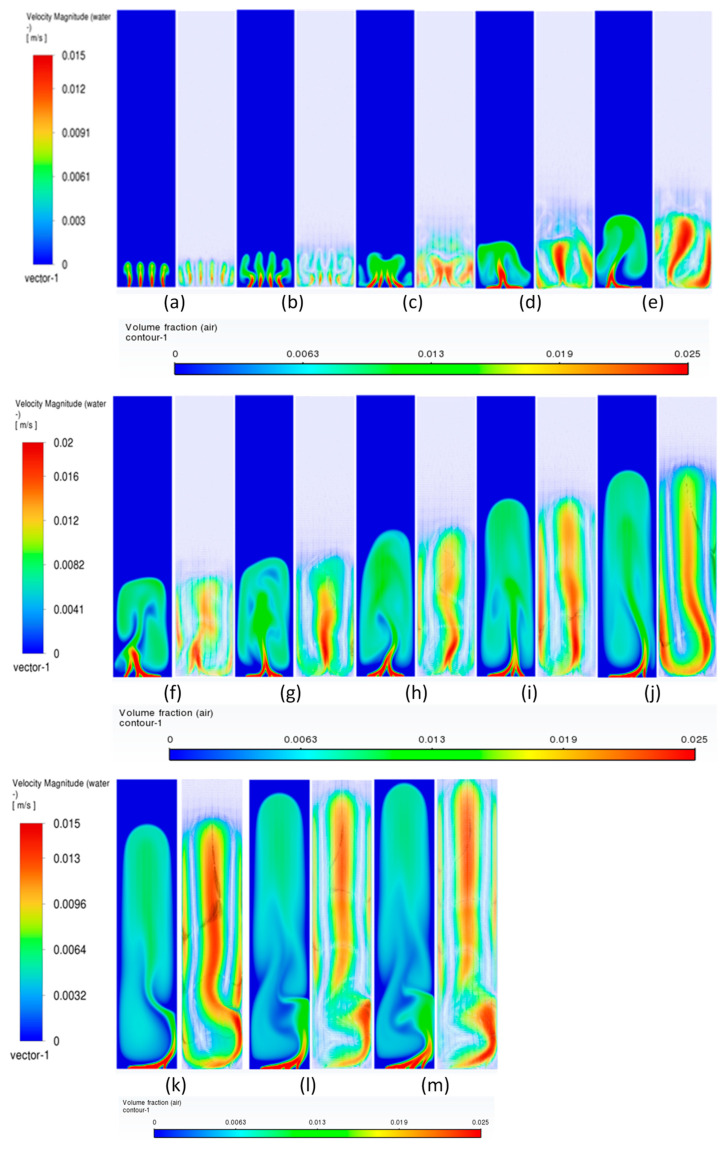
Instantaneous volume fractions and velocity vectors depicting flow structures in the gas–liquid bubble column with a hole diameter of 200 µm and a superficial gas velocity of 0.006 m/s for the central plane at Y = 0.025 m. (**a**) t = 10 s (**b**) t = 20 s (**c**) t = 30 s (**d**) t = 40 s (**e**) t = 50 s (**f**) t = 60 s (**g**) t = 70 s (**h**) t = 80 s (**i**) t = 90 s (**j**) t = 100 s (**k**) t = 110 s (**l**) t = 120 s (**m**) t = 123 s.

The flow patterns of gas for 0.004 m/s are similar to those of 0.002 m/s. However, some of the differences include the breaking of the mushroom structures multiple times. For example, the first mushroom structure develops between 60 and 80 s (Figure 12f–h) and disintegrates at 80 s with the upper part of the structure forming another mushroom structure while the other part at the bottom promotes mixing by forming re-circulatory cells. The second mushroom structure forms and disintegrates between 90 and 110 (Figure 12i–k) seconds, while the third structure starts forming from 110 s and ends at 130 s (Figure 12m). The repetitive pattern follows till the outlet. The flow structures, however, change at the superficial gas velocity of 0.006 m/s (Figure 13). Though the mushroom structure starts forming from 40 to 60 (Figure 13d–f) seconds, the disintegration does not happen, and a tree-shaped structure is formed with the plume at the vertically central part. With progress in time, the tree-shaped structure breaks with the lower section being well mixed, while the upper section maintains the tree-shaped structure, continuing for the upper section. At the end of steady state, mixing is observed in around 70% of the column.

#### 3.3.3. Plume Dynamics

**Figure 14:**
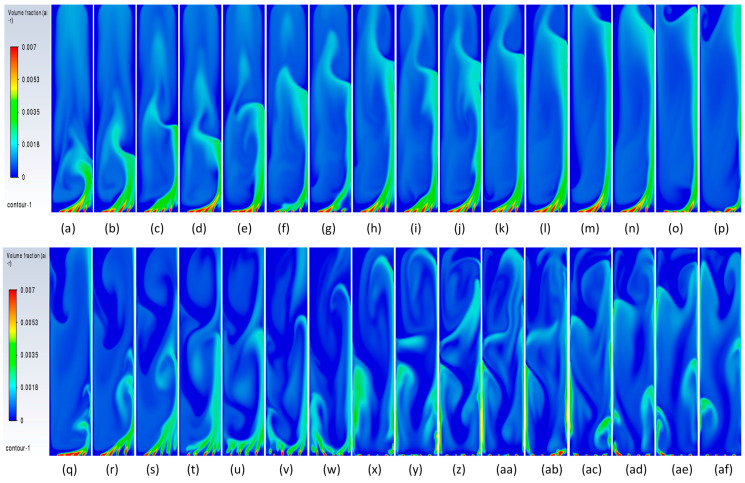

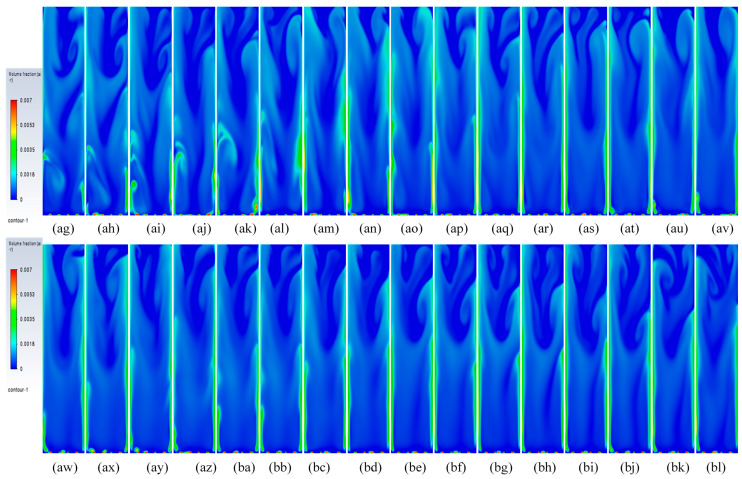
Instantaneous volume fractions depicting predictions of gas dynamics in the gas–liquid bubble column with a hole diameter of 200 µm and a superficial gas velocity of 0.002 m/s for the central plane at Y = 0.025 m. The start time is (**a**) t =130 s with a time gap of 10 s with (**bl**) = 730 s.

**Figure 15:**
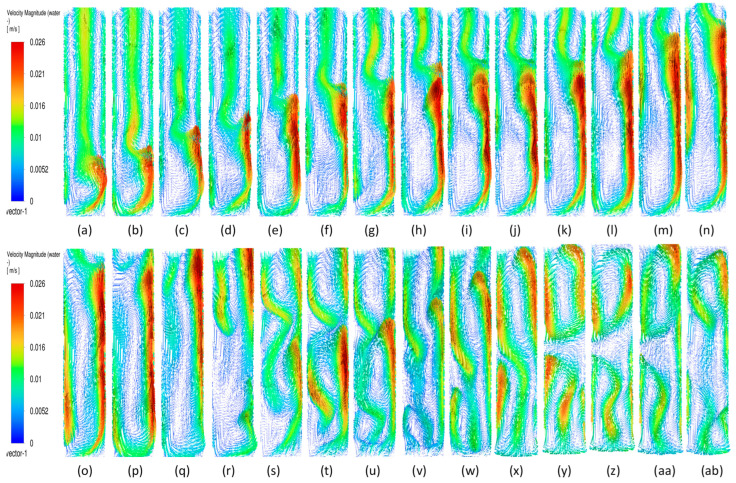

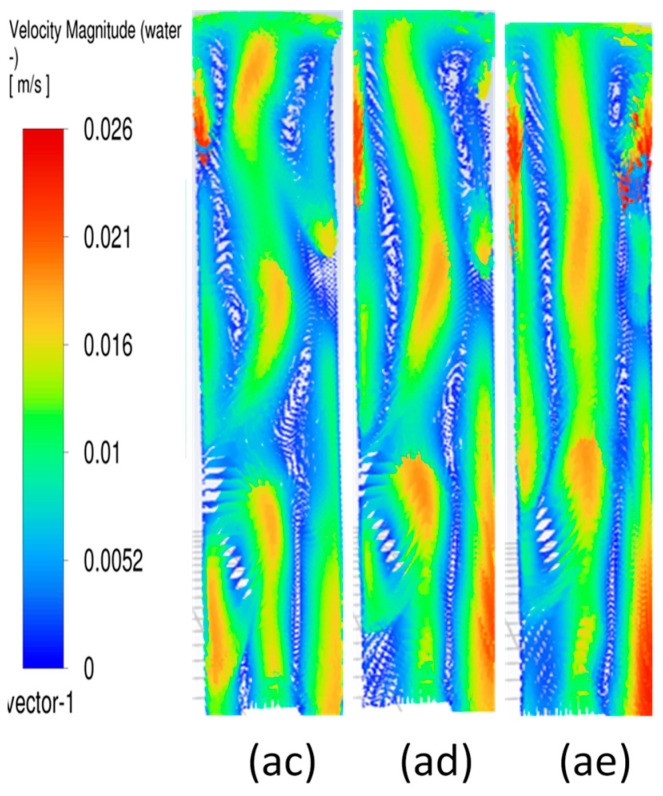
Instantaneous liquid velocity vectors depicting liquid dynamics in the gas–liquid bubble column with a hole diameter of 200 µm and superficial gas velocity of 0.002 m/s for the central plane at Y = 0.025 m. The start time is (**a**) t =130 s with a time gap of 10 s with (**ae**) = 430 s.

**Figure 16:**
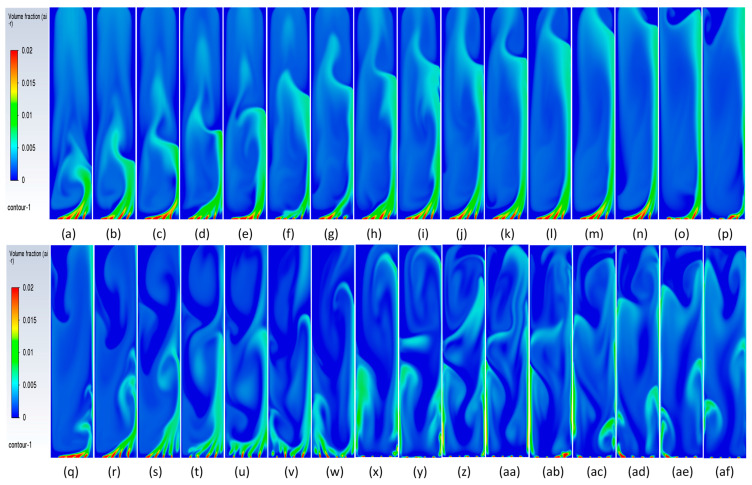

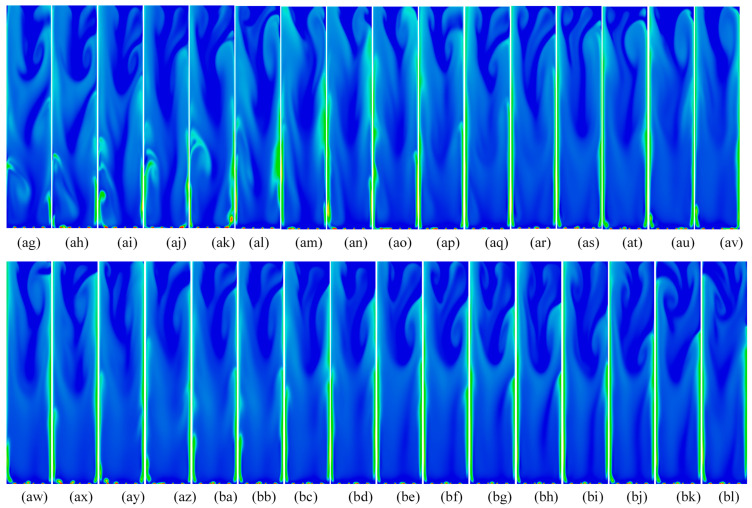
Instantaneous volume fractions depicting predictions of gas dynamics in the gas–liquid bubble column with a hole diameter of 200 µm and a superficial gas velocity of 0.004 m/s for the central plane at Y = 0.025 m. The start time is (**a**) t =130 s with a time gap of 10 s with (**bl**) = 730 s.

In this section, we discuss specifically the dynamics of the plume after it has finished its initial development till the gas reaches the top. Juliá et al. [16] have carried out comprehensive studies of gas movement through full and partial aeration when the number of holes is decreased from the sides to the center. However, the sparger type had a high number of holes, 135 < N < 25, where the maximum hole number represents all holes, while the minimum hole number shows the holes in the center. The authors defined three zones in which the central zone for airflow was termed Zone I, the formation of recirculation cells was termed Zone II, and the part of the column where the gas due to the gas moving towards the center was defined as Zone II. In the present work, we also define two zones: (a) a full aerated zone and (b) a partial aerated zone. However, while in the full aeration case the plume is static and rises straight with a parabolic profile at the center of axial liquid velocity and downward velocity near the walls, the partial aeration has two stages. The first stage involves the gas bubbles moving towards the right wall up to a certain distance and moving out from the corner. Initially, till about 300 s, while in the second stage, after 500 s, the bubbles move from the center holes in a straight line till the 50% height and then move right and leftwards, going out from the sides. Due to this, partial aeration is seen in the central zone in the form of a U-shape. The gas volume fraction is slightly higher in the wall region, with a distribution of gas in the rest of the region. During the first stage of partial aeration, the gas bubble moves away from the center towards the wall up to a certain distance due to the liquid displacing the bubbles to move away from the center. However, interestingly, the plume moves towards the right wall with an increase in time. The distance of the plume creeping upwards along the wall increases, after which it breaks and moves away from the wall towards the center and moves out. The plume is static but involves formation and breakage. A dynamic pattern is then developed, with gas layers in the center and sides as observed in Figure 14 and Figure 15 after 300 s moving upwards. This repeats every 10 s. However, this cannot be termed as plume oscillation frequency since the plume does not oscillate as it does in the case of centrally located spargers. Hence, Strouhal and Rayleigh number investigations have not been carried out. However, in Figure 15, from 420 s to 440 s, the plume oscillates and becomes straight, showing signs of oscillations every 10 s. A detailed study on this phenomenon is needed, which will be carried out further in future works. A similar sequence of flow patterns is observed for gas volume fraction in Figure 16 for the superficial velocity of 0.004 m/s and hole size of 200 μm. Since the patterns are similar, the ones of 0.006 m/s are not included.

**Figure 17:**
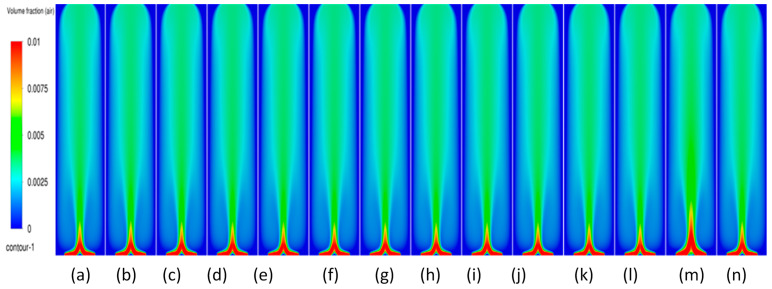
Instantaneous volume fractions depicting gas dynamics in the gas–liquid bubble column with a hole diameter of 600 µm and a superficial gas velocity of 0.002 m/s for the central plane at Y = 0.025 m. The start time is (**a**) t =80 s with a time gap of 10 s with (**n**) = 220 s.

**Figure 18:**
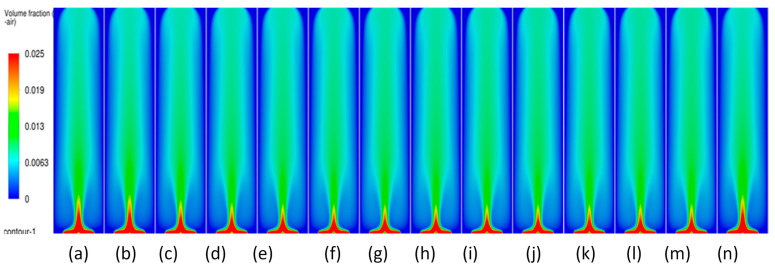
Instantaneous volume fractions depicting flow structures in the gas–liquid bubble column with a hole diameter of 600 µm and a superficial gas velocity of 0.004 m/s for the central plane at Y = 0.025 m. The start time is (**a**) t = 65 s with a time gap of 10 s with (**n**) = 205 s.

Figure 17 and Figure 18 show the sequence of the stable plume till 660 s and 226 s for two superficial velocities and a 600 μm hole size. This confirms the fact that the plume dynamics are different when the hole size is smaller than 200 μm for the same superficial velocities.

As per [40], the stability for uniform bubbly flow depends on the lower-order kinetics wave velocity. It was found that for all hole sizes and velocities considered for steady, the criteria are satisfied. However, transient numerical simulations have shown that for superficial velocities of 0.002 m/s and 0.006 m/s for a hole size of 200 µm, the flows are unstable for a certain period with dominant flow towards the right wall. This phenomenon needs to be studied in detail and is beyond the scope of the current study. The simulations have been carried out with considerable rigor, and reliability has been ensured. However, experimentation and simulation with LES would be taken up in the future.

### 3.4. Quantitative Analysis: Effect of Sparger Hole Size on Radial Profiles of Volume Fraction, Axial Liquid Velocity, and Turbulent Kinetic Energy (TKE)

In this section, instantaneous radial profiles of three quantities—volume fraction of the gas, axial velocity of the liquid, and TKE—have been analyzed for both hole diameters under study. Three superficial velocities (v_s_ = 0.002 m/s, 0.004 m/s, and 0.006 m/s) and three axial locations (z = 0.1 m, 0.25 m, and 0.4 m) have been chosen for each of the superficial velocities.

#### 3.4.1. Sparger Hole Size of 600 µm

**Figure 19:**
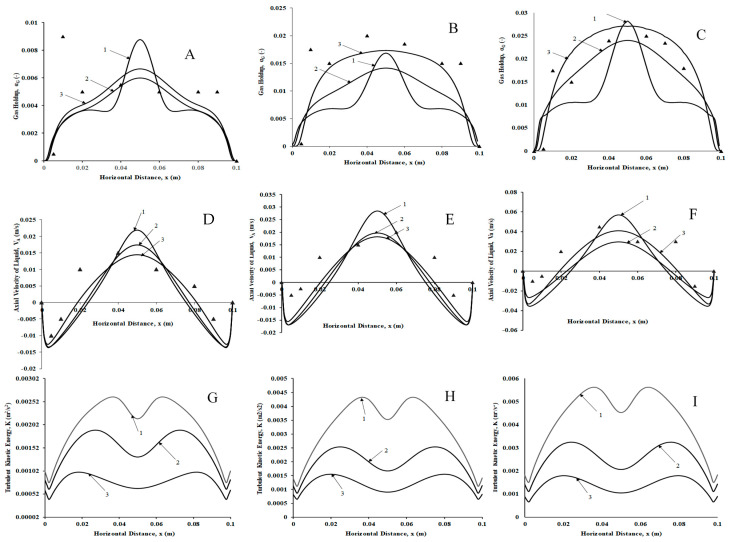
Time averaged radial profiles of volume fraction of the gas, liquid velocity, and turbulent kinetic energy for different superficial velocities for a hole size of 600 µm ((**A**,**D**,**G**) 0.002 m/s; (**B**,**E**,**H**) 0.004 m/s; (**C**,**F**,**I**) 0.006 m/s) at three different axial positions (1. Z = 0.1 m 2. Z = 0.25 m and 3. Z = 0.4 m). Triangle symbols denote experimental data of Sommer et al. [22].

Figure 19A–C shows the gas volume fraction profiles for three superficial velocities (v_s_ = 0.002 m/s, 0.004 m/s, and 0.006 m/s, respectively, each at z = 0.1 m, 0.25 m, and 0.4 m). Clearly, with an increase in axial location, the volume fraction profile changes from the bell-shaped curve for z = 0.1 m to a flat profile for z =0.4 m, as can be seen in Figure 19A. The bell-shaped curve denotes, for z = 0.1 m, the plume in the center, a flat region after which the holdup values decrease linearly, reducing to zero at the wall. The flat region is where the circulation cell of liquid with low liquid circulation velocities and constant gas holdup exists. However, for z = 0.25 m, the height of the bell-shaped region decreases, denoting the plume reaching its maximum height, while for z = 0.4 m, an overall flat velocity profile similar to the velocity profile in a turbulent pipe is observed, suggesting uniform bubble size distribution at the central axial height and above. This denotes that the lower position of the column has different hydrodynamics than the upper portion, where the gas holdup is uniform. Figure 19B shows a similar result, the difference being that the bell shape is restricted to z = 0.1 m, while the profile becomes near parabolic at z = 0.25 m and is less flat at z = 0.4 m than the case with a superficial gas velocity of 0.002 m/s. Finally, Figure 19C shows parabolic profiles for both z = 0.25 and 0.4 m, with a bell-shaped profile for z = 0.1 m. The predictions of z = 0.4 m also match very well (with a deviation of 15%) with the experimental data of Sommer et al. [22]. The probable reason for the deviation is due to the selection of the k-ε turbulence model.

The liquid axial velocity profiles for all three superficial velocities show an upward velocity at the center and a downward velocity near the walls (Figure 19D–F). The peak magnitudes of the liquid velocities increased as the superficial gas velocity increased, with the highest velocity peak for z = 0.1 m. For the other two axial positions, the velocity magnitudes and the profile trends are almost similar. The upward velocity is maximum when the liquid is carried upward by the high gas holdup plume and decreases to zero on both ends at approximately 50% column width after flow inversion occurs. This is called the inversion point, after which velocities move in a downward direction with a peak having a magnitude similar to the peak of z = 250 and 0.4 m widths. The predictions of z = 0.4 m also match very well (with a deviation of 5–10%) with the experimental data of Sommer et al. [22].

The TKE profiles (Figure 19G–I) show an inverted bell shape for all superficial velocities, as have also been reported by Deen et al. [47], in their experimental investigations. However, turbulence in the present case is low compared to the reported literature [47] in the liquid phase since superficial velocities are significantly (~two orders of magnitude) lower.

#### 3.4.2. Sparger Hole Size of 200 µm

**Figure 20:**
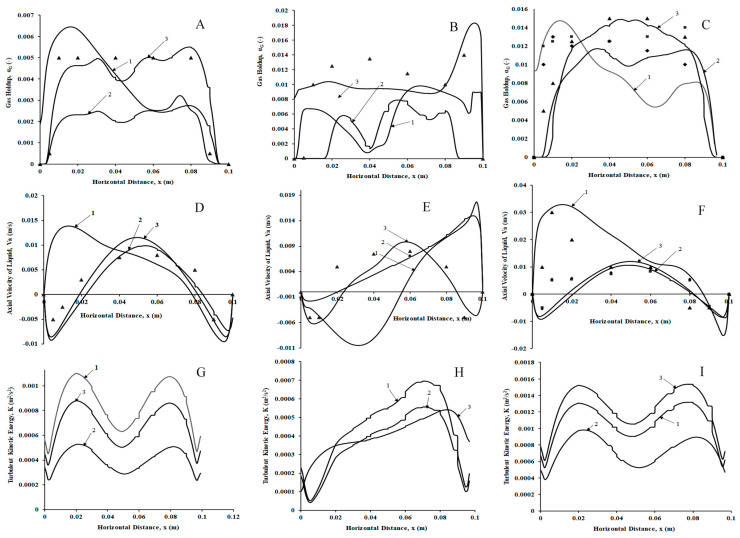
Time-averaged radial profiles of volume fraction of the gas, liquid velocity, and turbulent kinetic energy for a hole size of 200 µm and different superficial velocities ((**A**,**D**,**G**) 0.002 m/s; (**B**,**E**,**H**) 0.004 m/s; (**C**,**F**,**I**) 0.006 m/s) at three different axial positions (1. z = 0.1 m, 2. z = 0.25 m, and 3. z = 0.4 m) of 200 µm. The symbols denote experimental data of Sommer et al. [22].

Figure 20A–C shows the time-averaged gas volume fraction profiles for three superficial velocities (v_s_ = 0.002 m/s, 0.004 m/s, and 0.006 m/s, respectively, each at z = 0.1 m, 0.25 m, and 0.4 m for a 200 µm hole size). As shown in Figure 20A, for a lower superficial gas velocity of 0.002 m/s, the volume fraction profiles are skewed and asymmetric with a peak away from the center and nearer to the left wall for z = 0.1 m. For the other two axial locations, flat gas volume fraction profiles can be observed, denoting uniform mixing at the upper portion of the column. Figure 20B shows the time-averaged gas volume fraction profiles for a superficial gas velocity of 0.004 m/s and the three axial locations considered for the study. The gas volume fraction profiles denote that at z = 0.1 m, the gas volume fraction is absent at nearer regions near the wall, while a complex nonlinear profile exists in the central region at this location. At z = 0.25 m, however, a flat volume fraction profile is seen in the entire region except near the walls (Figure 20B, legend 2). For z = 0.4 m (Figure 20C), a parabolic profile is seen at z = 0.4 m and a flat profile at z = 0.25 m, while a nonlinear profile is observed with higher volume fractions near the left wall and lower volume fractions near the center and right wall. The predictions for all axial positions with the experimental data of Sommer et al. [22] are shown in Figure 20C. While the predictions match well with a deviation of 5–10% for the axial positions z = 0.25 m and 0.4 m, the deviations are high for z = 0.1 m. While the trends are similar, the velocities are under-predicted in the positions from 0.030 m to 0.065 m. While this fact is intriguing, the under-predictions are found to be better than the model predictions of Sommer et al. [22]. Further, the present model is able to predict the trend of the profile, which could not be found in Sommer et al. [22].

The liquid axial velocity profiles for the three superficial velocities and different locations also complement the observations of gas volume fraction profiles. For example, Figure 20D,F show the upward velocities at the center and downward velocities near the walls for z = 0.25 m and z = 0.4 m. However, the radial velocity profiles are different at z = 0.1 m for both cases. The axial velocities are upward and higher in magnitude near the left wall and go at an inversion point at z = 0.080 m, where the downward velocities are observed for 0.002 m/s and at z = 0.090 m for 0.006 m/s. However, for a superficial gas velocity of 0.004 m/s, the trends are different at each axial location, as can be observed in Figure 20E. A clear asymmetry is observed in all the locations. For z = 0.1 m, the upward velocities at the center and downward velocities near the walls are observed. However, for z = 0.25 m downward velocities near the left wall, an inversion point at z = 0.020 m, and upward velocities in the remaining region with a linear increase with radial distance till the right wall is encountered are observed. For z = 0.4 m, however, the inversion point occurs at z = 0.06 m with upward velocities having higher magnitudes than downward velocities. It has also been observed that both the profile trends and the magnitudes match well with the experimental data of Sommer et al. [22] with a deviation of less than 10% for all the axial positions for the superficial gas velocity of 0.006 m/s, as observed in Figure 20G.

The TKE profiles (Figure 20G,I) represent a sinusoidal profile for both 0.002 m/s and 0.006 m/s. The trends in the profile are similar to the ones observed in the literature, Deen et al. [47]. Figure 20H shows slightly different profiles with higher values in the central region for z = 0.1 m, while for the other two locations, a skewed profile (with a peak toward the right wall) is observed.

It should be noted that a decrease in hole size has an impact on the liquid axial velocities for the same bubble size and superficial gas velocities. The liquid axial velocities are in the range of 3 times the superficial gas velocities, while the gas volume fractions are slightly less than two orders of magnitude lower than the original gas volume fractions reported by the experimental studies of Sommer et al. [22].

## 4. Empirical Correlations for Gas Holdup and Liquid Velocity

**Figure 21:**
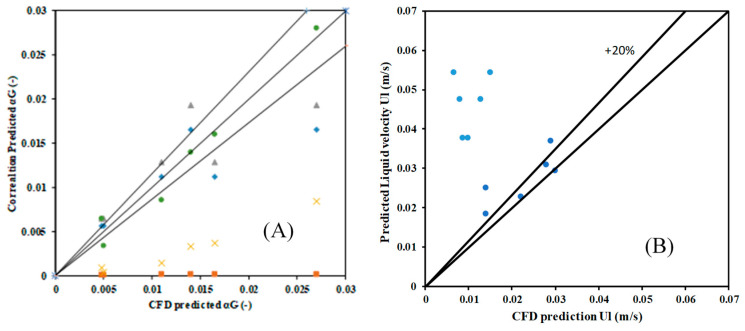
A parity plot of predictions of the present work with correlations obtained for similar operating conditions in the literature: (**A**) gas holdup grey triangle, light blue rhombus, yellow cross, and orange square denote correlation predictions of Hughmark [32]; Kato [56]; Sal et al. [38] and Kato et al. [57], respectively, while circles denote correlation of the present work (**B**) liquid velocity. light blue solid circles, Zehner [44]; dark blue circles, solid line present work.

From the gas holdup data obtained from the CFD simulations, the following correlations have been obtained.

(24) $$\alpha_{G}=5.735{\left({U_{g}}^{2}/d_{o}g\right)}^{0.67}{\left({d_{o}}^{2}\rho_{l}g/\sigma \right)}^{0.63}{\left(z/H\right)}^{-0.03}$$

(25) $$U_{l}=0.12{\left(\alpha_{G}\right)}^{0.33}$$

where Equation (24) denotes the empirical correlation for gas holdup, while Equation (25) represents the correlation for liquid axial velocity. Figure 21A shows the parity plot of gas holdup while Figure 21B shows the parity plot of liquid velocity predicted by different correlations (including the one of the present work) compared with the data obtained from the predicted CFD model.

The present correlation was developed in view of the fact that, by careful investigation of the dimensionless groups that had the most significant influence on the gas holdup. All correlations from the literature are chosen by considering the fact that the superficial velocities are the same. The present correlation predictions for gas holdup are in good agreement with the simulated data. The correlations of Kato et al. [57] and one of Hughmark [32] predict well with deviations around 25%, while the predictions of Sal et al. [38] and Kato [56] show very high deviations (>100%). This is typically due to the dependence of data on the distributor design and the range of superficial velocities chosen. Similar observations can be observed for liquid velocity (Equation (25)). Figure 21B shows the parity plot of liquid velocity comparisons of predictions by the present correlation and the ones available in the literature. While the correlation developed in the present work shows deviations of ~20%, the other correlations show deviations of (>100%). This is attributed to the fact that the present data is a strong function of gas holdup, whereas other correlations are a function of multiple dimensionless groups, including gravity and geometric parameters.

## 5. Conclusions

With a focus on characterizing a bubble column with a uniquely designed uniform sparger, the flow across two hole diameters and three superficial velocities was investigated. The following conclusions can be drawn regarding flow patterns:

1. Both gas and liquid flow patterns are observed to be different for the present sparger design, especially for a hole size of 200 μm for all the superficial velocities than the conventional bubble column flow patterns reported in the literature.
2. A steady static fully aerated bubble plume is observed for a hole size of 600 μm for all the superficial velocities investigated after a certain time. However, the plume develops initially with the gas from all holes moving to the center and combining to form a plume.
3. For a hole size of 200 µm, a dynamic flow is observed in three stages: plume development, wall-sided plume movement, and subsequent upward movement of the gas plume with five alternating straight lines of high and low liquid velocities. A vortical structure is observed in the upper 0.15 m, with a region comprising mostly liquid and gas moving from the sides after a time of 500 s.
4. During the initial plume development for both hole sizes and all superficial velocities, different shapes are observed resembling shapes similar to those found in nature, and several vortical structures involving good mixing. This might help in achieving fast reactions only in the time frames when these shapes are formed.
5. The gas holdup profiles for a hole size of 600 µm show a bell-shaped curve for an axial distance of 0.1 m, while the profiles become parabolic till z = 0.4 m, showing a demarcation in the lower and upper zones. For a 200 µm hole size, however, the gas holdup profiles have a different pattern at z = 0.1 m. However, for z = 0.25 m and z = 0.4 m, it becomes parabolic for all superficial velocities, representing homogeneous flow at the top portion.
6. Bell-shaped instantaneous radial profiles for axial velocities are observed, characterized by upward velocities in the plume region and downward velocities near the wall for all superficial velocities for the hole size of 600 µm. With an increase in the axial height, the magnitudes of axial velocities decrease. The radial velocity profiles of liquid axial velocities differ for z = 0.1 m to z = 0.25 m and z = 0.4 m. For the hole size of 200 µm, the velocities are skewed towards the right or left wall, depending on the axial position, and in the upward direction for all superficial velocities investigated.
7. TKE radial profiles demonstrate that, though low in magnitude, they have the potential to promote mixing in the lower part of the column for all superficial velocities considered and a hole size of 200 µm. For a higher hole size, a parabolic profile with a dip at the center is observed, demonstrating jetting flow for all operating conditions and axial positions.

In summary, it is observed that the hole size of 600 µm gives relatively lower mixing than the hole size of 200 µm due to the formation of stronger, smaller circulation cells as well as small flow structures (plume dynamics). The most significant contribution of this work is the fact that mixing can be obtained even at low superficial velocities, which can serve for contacting equipment where shear needs to be minimized.

## Data Availability

Data is confidential but can be made available on the genuineness of the requirement.

## Nomenclature

*b*	[m]	Minor radius of bubble
*C*	[–]	Coefficient
*C_D_*	[–]	Drag coefficient
*C_D,sphere_*	[–]	Drag correlation for spherical shape
*C_D,ellipse_*	[–]	Drag correlation for ellipsoidal shape
*C_D,cap_*	[–]	Drag correlation for cap shape
*C*	[–]	Empirical constant in radial profile
*d*	[m]	Diameter of holes
*d_b_*	[m]	Bubble diameter
*d_o_*	[m]	Orifice diameter
*d_32_*	[m]	Sauter mean diameter of bubble
*D*	[m]	Column diameter
*E*	[–]	Uncertainty
*Eo*	[–]	Eötvös number
*f*	[–]	Empirical factor (1 for pure liquids)
*F*	[N m^−3^]	Force per unit volume
*g*	[m s^−2^]	Acceleration of gravity
*I*	[–]	Bubble object
*IoU*	[–]	Intersection over union
*k*	[–]	Empirical constant (from [46])
*k*	[m^2^ s^−1^]	Turbulent kinetic energy
*k_L_*	[m^2^ s^−1^]	Turbulent kinetic energy in the liquid
*K_d_*	[–]	Empirical constant
*n*	[–]	Empirical constant (from [43])
*N*	[–]	Number of holes
*p_t_*	[Pa]	Total pressure
*p_s_*	[Pa]	Saturation pressure
*p*	[Pa]	Pressure
*P*	[–]	Persistence
*Q*	[L min^−1^]	Flow rate
*Q_g_*	[L min^−1^]	Gas flow rate
*Re_B_*	[–]	Bubble Reynolds number, dimensionless
*Re*	[–]	Reynolds number
*s*	[–]	Standard deviation
*t*	[s]	Time
*U_g_*	[m s^−1^]	Superficial gas velocity
*U_l_*	[m s^−1^]	Superficial liquid velocity
*U_l,max_*	[m s^−1^]	Maximum liquid velocity
*U_df_*	[m s^−1^]	Minimum fluidization gas velocity
*U′*	[–]	Dimensionless velocity parameter
*u_rel_*	[m s^−1^]	Relative velocity
*u*	[m s^−1^]	Phase velocity
*u_G_*	[m s^−1^]	Gas phase velocity
*u_L_*	[m s^−1^]	Liquid phase velocity
*V*	[m^3^]	Volume
*V_a_*	[m s^−1^]	Velocity of gas
*V_f_*	[m s^−1^]	Fluid velocity
*We*	[–]	Weber number
x	[m]	Horizontal distance
Greek Letters		
*α_g_*	[–]	Gas holdup
*α_g,SB_*	[–]	Gas holdup due to small bubbles
*α_g,LB_*	[–]	Gas holdup due to large bubbles
*α_gm_*	[–]	Transitional gas holdup
*λ*	[nm]	Wavelength
*μ*	[kg s^−1^m^−1^]	Dynamic viscosity
*μ_l_*	[kg s^−1^m^−1^]	Dynamic viscosity of liquid
*μ_g_*	[kg s^−1^m^−1^]	Dynamic viscosity of gas
*μ_t,L_*	[kg s^−1^m^−1^]	Turbulent viscosity in the liquid
*ρ*	[kg m^−3^]	Density
*ρ_l_*	[kg m^−3^]	Liquid density
*ρ_g_*	[kg m^−3^]	Gas density
*ρ_i_*	[kg m^−3^]	Intermediate or phase density (context-dependent)
*σ*	[N m^−1^]	Surface tension
*τ*	[Pa]	Stress tensor
*τ_G_*	[Pa]	Viscous stress tensor for gas
*τ_L_*	[Pa]	Viscous stress tensor for liquid
*Δ*	[–]	Delta
*Δρ*	[kg m^−3^]	Density difference, ρ_L_ − ρ_G_
*ε_L_*	[m^2^ s^−1^]	Turbulent dissipation rate in the liquid
*ϕ*	[–]	Scaler field
*ξ*	[–]	Dimensionless radial co-ordinates
*ξ_inv_*	[–]	Radial position of flow inversion
*Γ*	[–]	Empirical correlation parameter
Sub- and Superscripts		
*B*	Bubble	
*b*	bubble	
*cal*	Calibration	
*cap*	Cap-form bubble	
*crit*	Critical	
*disp*	Displacement	
*Ed*	Euclidian	
*eff*	Effective	
*ellipse*	Ellipsoidal	
*exp*	Exponential	
*G*	Gas phase	
*gt*	Ground truth	
*i*	Inner	
*inter*	Interfacial	
*L*	Liquid phase	
*mol*	Molecular	
*max*	Maximum	
*pred*	Predicted	
*ref*	Reference	
*rel*	Relative	
*sEd*	Sum of successive Euclidian	
*sphere*	Spherical bubble	
*turb*	Turbulent	
*TD*	Turbulent dispersion	
*w*	Wall	
Abbreviations		
CFD	Computational Fluid Dynamics	
EE	Euler–Euler	
EL	Euler–Lagrangian	
PBM	Population Balance Method	
EE-PBM	Euler–Euler with Population Balance Method	
RANS	Reynolds-averaged Navier–Stokes	
LES	Large Eddy Simulation	
BCR	Bubble column reactor	
BC	Bubble column	
